# Acetylcholine receptor-β inhibition by interleukin-6 in skeletal muscles contributes to modulating neuromuscular junction during aging

**DOI:** 10.1186/s10020-024-00943-3

**Published:** 2024-10-10

**Authors:** Yanling Zhao, Han Yan, Ke Liu, Jiangping Ma, Wenlan Sun, Hejin Lai, Hongli Li, Jianbang Gu, He Huang

**Affiliations:** 1 Department of Geriatrics, Shanghai General Hospital, Shanghai Jiao Tong University School of Medicine, Shanghai, 200080 P.R. China; 2 Department of Neurology, Tenth People’s Hospital, Tongji University School of Medicine, Shanghai, 200072 P.R. China; 3 CAS Key Laboratory of Nutrition, Metabolism and Food Safety, Shanghai Institute of Nutrition and Health, University of Chinese Academy of Sciences, Chinese Academy of Sciences, Shanghai, 200031 China; 4 Department of Neurology, Shanghai Tenth People’s Hospital Chongming Branch, Shanghai, 202150 P.R. China

**Keywords:** Neuromuscular junction, Interleukin-6, Acetylcholine receptor β, Peroxisome proliferator-activated receptor gamma coactivator 1α, Myocyte enhancer factor 2 C, Aging

## Abstract

**Background:**

Aging-related strength decline contributes to physiological deterioration and is a good predictor of poor prognosis. However, the mechanisms underlying neuromuscular junction disorders affecting contraction in aging are not well described. We hypothesized that the autocrine effect of interleukin (IL)-6 secreted by skeletal muscle inhibits acetylcholine receptor (AChR) expression, potentially causing aging-related strength decline. Therefore, we investigated IL-6 and AChR β-subunit (AChR-β) expression in the muscles and sera of aging C57BL/6J mice and verified the effect of IL-6 on AChR-β expression.

**Methods:**

Animal experiments, in vitro studies, bioinformatics, gene manipulation, dual luciferase reporter gene assays, and chromatin immunoprecipitation experiments were used to explore the role of the transcription cofactor peroxisome proliferator-activated receptor gamma coactivator 1-α (PGC1α) and its interacting transcription factors in the IL-6-mediated regulation of AChR-β expression.

**Results:**

IL-6 expression gradually increased during aging, inhibiting AChR-β expression, which was reversed by tocilizumab. Both tocilizumab and the PGC1α agonist reversed the inhibiting effect of IL-6 expression on AChR-β. Compared to inhibition of signal transducer and activator of transcription 3, extracellular signal-regulated kinases 1/2 (ERK1/2) inhibition suppressed the effects of IL-6 on AChR-β and PGC1α. In aging mouse muscles and myotubes, myocyte enhancer factor 2 C (MEF2C) was recruited by PGC1α, which directly binds to the AChR-β promoter to regulate its expression.

**Conclusions:**

This study verifies AChR-β regulation by the IL-6/IL-6R-ERK1/2-PGC1α/MEF2C pathway. Hence, evaluating muscle secretion, myokines, and AChRs at an earlier stage to determine pathological progression is important. Moreover, developing intervention strategies for monitoring, maintaining, and improving muscle structure and function is necessary.

**Supplementary Information:**

The online version contains supplementary material available at 10.1186/s10020-024-00943-3.

## Background

Sarcopenia, characterized by an aging-related decline in skeletal muscle mass, muscle strength, and/or physical performance, is responsible for the physiological deterioration associated with aging (Cruz-Jentoft et al. 2019a, b). Older people use different motor unit activation strategies during knee extension to generate the same amount of force as younger people, thereby concealing muscle strength changes and preventing early detection of physiological deterioration (Ling et al. 2009). At a single-fiber level, the decline in strength is larger than the decline in muscle mass and occurs earlier (Ling et al. 2009; Ferrucci et al. 2012). The muscle mass gain cannot prevent the age-related decline in strength (Goodpaster et al. 2006). In the definition of sarcopenia revised by the European Working Group on Sarcopenia in Older People in early 2018, the decrease in muscle strength, rather than the decrease in muscle mass, is the primary factor determining parameters of sarcopenia (Cruz-Jentoft et al. 2019a, b).

The typical pathology of sarcopenia is muscle atrophy and muscle loss, characterized by denervation-like changes thought to be due to motoneuron degeneration (Gustafsson and Ulfhake 2021). However, changes in the neuromuscular junction (NMJ), which mediates the excitation–contraction coupling between the nerve and muscle, emerge earlier during the disease (Larsson et al. 2019; Ferraro et al. 2012). During the early aging stages, no notable atrophy of muscle fibers or changes in NMJ presynaptic and postsynaptic membranes emerge; however, the axoplasmic transport of motor nerves is altered. A decline in the expression of muscle-derived trophic signals, such as brain-derived neurotrophic factors and C-terminal agrin fragments, leads to disorders of axoplasmic transport and imbalances in denervation–reinnervation (Marzetti et al. 2014; Moreira-Pais et al. 2022). Hence, changes in the NMJ in early aging precede nerve degeneration and muscle atrophy (Alchin 2014). Acetylcholine receptors (AChR) are indispensable for NMJ development, stabilization, and plasticity (Tintignac et al. 2015; Wang et al. 2018). Given that the postsynaptic domain appears to differentiate before innervation in the center of the muscle via pre-patterning (Darabid et al. 2014), AChR-NMJ alterations are believed to be pathologically intrinsic to aging rather than a consequence of motoneuron degeneration (Blasco et al. 2020).

Chronic low-grade inflammation (LGI) is a low-grade and sterile inflammatory state that occurs with aging and is a major contributing factor to the pathogenesis of sarcopenia and other aging-related diseases (Fulop et al. 2018). Skeletal muscle cells, as endocrine cells, secrete pro-inflammatory factors (called myokines), such as interleukin (IL)-6 and myostatin, to affect their function and that of multiple organs (Severinsen and Pedersen 2020; Fu et al. 2020). Under LGI conditions, IL-6 influences the synthesis and degradation of contraction-related proteins through signal transducer and activator of transcription 3 (STAT3) and extracellular signal-regulated kinases 1/2 (ERK1/2) pathways in skeletal muscles (Forcina et al. 2022). However, the effects and potential mechanisms of IL-6 on AChRs remain unclear.

The AChR β-subunit (AChR-β) plays a crucial role in agrin-induced cytoskeleton anchoring, aggregation, and stable clustering of AChRs (Rudell and Ferns 2013), making it an indispensable component and a rate-limiting factor for maturation of the NMJ (Joassard et al. 2015). In contrast to increasing of AChR-ɑ and -γ mRNA expression in older women compared with young women, AChR-β mRNA decreases with aging (Soendenbroe et al. 2020). In this study, we focused specifically on AChR-β and explored the effects of IL-6 on AChR-β expression in skeletal muscles during aging to elucidate the possible mechanisms involved in the decline in muscle strength.

## Methods

Unless otherwise stated, all reagents were purchased from Sinopharm Chemical Reagents (Shanghai, China).

### Preparation of animal models and acquisition of specimens

Skeletal muscle strength (as assessed by grip strength) of C57BL/6J mice increased continuously until the mice were 10 months old, and muscle strength began declining from 13 months old (Xie et al. 2021; Dijk et al. 2011, 2017). In contrast, muscle mass did not decrease until 16 months old. Therefore, 15-month-old early-aging mice were used in this study to avoid muscle atrophy (Dijk et al. 2017). Eight-week-old (adult), 15-month-old (early aging), and 20-month-old (aging) C57BL/6J mice were purchased from the Laboratory Animal Resources, Chinese Academy of Sciences (Shanghai, China). The mice were housed in a well-controlled environment (55 ± 5% humidity, 12-h day/12-h night cycles, and 25 ± 2 °C) at the Laboratory Animal Research Center of the Tenth People’s Hospital (Shanghai, China). All experimental procedures and protocols were reviewed and approved by the Animal Experimentation Ethics Committee of the Tenth People’s Hospital affiliated with Tongji University (SHDSYY-2020-2458).

The animals were divided into the following groups: adult, early aging, treatment control (early aging + phosphate-buffered saline [PBS]) group, treatment (early aging + drug treatment) group, and aging group. Each group comprised three males and three females (*n* = 6). The drug treatment group intraperitoneally received the IL-6 receptor antibody tocilizumab (HY-P9917, 100 µg/mL in PBS; MedChemExpress, South Brunswick Township, NJ, USA). For each mouse, 100 µL of drug solution was administered once every 48 h for 20 days. The control group was administered the same volume of PBS intraperitoneally.

After treatment (20 days of drug administration), the mice were euthanized by an intraperitoneal injection of 10% chloral hydrate (0.35 mL/100 g weight). The blood collected from the heart was centrifuged at 1500 ×*g* for 10 min at 4 °C to harvest the serum. Simultaneously, the extensor digitorum longus (EDL), soleus (SOL), and tibialis anterior (TA) muscles were harvested. Serum and muscles specimens were stored at − 80 °C.

### Cell culture and differentiation induction

The mouse myoblast cell line C2C12 was maintained in Dulbecco’s modified Eagle medium (DMEM) containing 10% fetal bovine serum (Gibco, Thermo Fisher Scientific, Waltham, MA, USA) and differentiated in DMEM supplemented with 2% horse serum (Gibco) after reaching a confluence of approximately 70–80%, as previously described (Huang et al. 2019). After six days, the differentiated C2C12 myotubes were used for subsequent experiments.

### Plasmid construction

The full-length complementary DNA (cDNA) of myocyte enhancer factor 2 C (MEF2C; synthesized by Sangon Biotech, Shanghai, China) was cloned into plasmid cloning DNA (pcDNA)3.1(+) myc-His A between EcoRI and XhoI sites to obtain pcDNA3.1-MEF2C plasmid. The plasmid was used to measure MEF2C overexpression. For the luciferase reporter assay, the AChR promotor sequence (from − 2000 bp to + 50 bp of the transcription initiation site), synthesized by General Biosystems, was cloned into pGL3 plasmid (Promega, Madison, WI, USA) between the MluI and SmaI sites. According to the manufacturer’s instructions, pGL3-AChR mutant luciferase reporter plasmids with mutations in the predicted binding region were constructed with QuikChange™ Site-Directed Mutagenesis Kit from Stratagene. The mutant sequences of the pGL3-AChR mutant reporter plasmids were as follows: pGL3-AChR mut1, 5′-CTGTTGTTATA-3′ to 5′-AAAAAAAAAAA-3′; pGL3-AChR mut2, 5′-TATAGACACAT-3′ to 5′-AAAAAAAAAAA-3′; pGL3-AChR mut3, 5′-TATAGTGACGA-3′ to 5′-AAAAAAAAAAA-3′.

### Cell transfection

For transfection of the overexpression plasmid, C2C12 myotubes in 12-well plates were transfected with the pcDNA3.1-MEF2C plasmid at a final concentration of 1 µg in 1 mL of the culture medium per well using Lipofectamine 3000 reagent (Invitrogen, Carlsbad, CA, USA).

For transfection of small interfering RNA (siRNA), C2C12 myotubes in 12-well plates were transfected with MEF2C siRNAs at a final concentration of 50 nM in 1 mL of the culture medium per well using Lipofectamine 3000 reagent. The siRNA sequences of MEF2C with a mixture of two oligos (synthesized by General Biosystems, Shanghai, China) were as follows: siMEF2C-1, 5′-UGGAUAAGGUGUUGCUCAA-3′ (dTdT) and 5′-UUGAGCAACACCUUAUCCA-3′ (dTdT); siMEF2C-2, 5′-GGAAAUUUGGAUUGAUGAA-3′ (dTdT) and 5′-UUCAUCAAUCCAAAUUUCC-3′ (dTdT); siMEF2C-3, 5′-CAAGAAUAUACAAGCCAAA-3′ (dTdT) and 5′-UUUGGCUUGUAUAUUCUUG-3′ (dTdT). The siMEF2C-2 was used in subsequent studies based on the observed effect of different siRNAs on MEF2C expression.

After plasmid or siRNA transfection for 48 h, the cells were harvested to extract total RNA or for western blotting.

### Enzyme-linked immunosorbent assay

Following the manufacturer’s instructions, IL-6 levels were quantified using a commercial enzyme-linked immunosorbent assay kit (PI326, Beyotime Biotechnology, Shanghai, China). The absorbance value was measured at 450 nm using a microplate reader.

### Protein extraction and quantification

Mouse muscle tissues were cut and placed in a crushing tube with 4–6 magnetic beads; pre-chilled RIPA buffer was added to the tube, immediately placed into the crusher, and crushed at 60 Hz for 10 min. The homogenate was centrifuged at 10,000 ×*g* and 4 °C for 20 min, and the supernatant was collected.

C2C12 myotubes were homogenized with a lysis buffer containing RIPA buffer and protease inhibitor cocktails (1:1000; Sigma-Aldrich, St. Louis, MO, USA) on ice before centrifugation (10 000 ×*g*, 10 min) to remove insoluble material. The protein concentration was determined using the Bradford assay with bovine serum albumin as the standard.

### Western blotting

Protein samples were analyzed with antibodies against IL-6 (1:1000, ab259341, Abcam, Cambridge, UK), AChR-β (1:500, sc-65813; Santa Cruz Biotechnology, Dallas, TX, USA), peroxisome proliferator-activated receptor gamma coactivator 1-α (PGC1ɑ; 1:2000, ab313559; Abcam, Cambridge, UK), and MEF2C (1:1000, ab211493; Abcam, Cambridge, UK). Each blot shown in the figures is representative of at least three experiments. Protein expression was detected via enhanced chemiluminescence (Merck KGaA, Darmstadt, Germany) and normalized against tubulin.

### Reverse transcription-polymerase chain reaction

Total RNA from muscles or C2C12 from myotubes was extracted using TRIzol reagent (19C30; Thermo Fisher Scientific) following the manufacturer’s instructions. RNA (1 µg) was used to perform first-strand cDNA synthesis and reverse transcription using a PrimeScript reagent kit (RR037A; TakaraBio, Shiga, Japan). Real-time polymerase chain reaction (PCR) analyses were performed using ABI 7900 (Roche, Basel, Switzerland) with FastStart Universal SYBR Green Master (Roche). Following initial denaturation at 95 °C for 2 min, the PCR conditions comprised 40 cycles at 95 °C for 10 s and 60 °C for 60 s. Relative gene expression was calculated using the 2^–ΔΔCt^ method and normalized against the housekeeping gene β-actin to compensate for variations in input cDNA. The PCR primers used in this study are listed in Table 1.

**Table 1:** qPCR primers used in this study

Primer name	Primer sequence (5ʹ → 3ʹ)
IL-6 Forward	TACCACTTCACAAGTCGGAGGC
IL-6 Reverse	CTGCAAGTGCATCATCGTTGTTC
AChR-β Forward	AAGTCAACCGCCACCTTCAGAC
AChR-β Reverse	GTAGCCAATCACAGTGTAGAGGG
PGC1ɑ Forward	GAATCAAGCCACTACAGACACCG
PGC1ɑ Reverse	CATCCCTCTTGAGCCTTTCGTG
MEF2C Forward	GTGGTTTCCGTAGCAACTCCTAC
MEF2C Reverse	GGCAGTGTTGAAGCCAGACAGA
MEF2D Forward	GGTTTCCGTGGCAACACCAAGT
MEF2D Reverse	GCAGGTGAACTGAAGGCTGGTA
ERR1 Forward	ACTACGGTGTGGCATCCTGTGA
ERR1 Reverse	GGTGATCTCACACTCATTGGAGG
PPAR-ɑ Forward	ACCACTACGGAGTTCACGCATG
PPAR-ɑ Reverse	GAATCTTGCAGCTCCGATCACAC
PPAR-γ Forward	GTACTGTCGGTTTCAGAAGTGCC
PPAR-γ Reverse	ATCTCCGCCAACAGCTTCTCCT
Hnf4ɑ Forward	TGCGAACTCCTTCTGGATGACC
Hnf4ɑ Reverse	CAGCACGTCCTTAAACACCATGG
GR Forward	TGGAGAGGACAACCTGACTTCC
GR Reverse	ACGGAGGAGAACTCACATCTGG
FOXO-1 Forward	CTACGAGTGGATGGTGAAGAGC
FOXO-1 Reverse	CCAGTTCCTTCATTCTGCACTCG
Actin Forward	CATTGCTGACAGGATGCAGAAGG
Actin Reverse	TGCTGGAAGGTGGACAGTGAGG

qPCR, quantitative polymerase chain reaction, AChR-β, Acetylcholine receptor β-subunit; IL, interleukin; MEF, myocyte enhancer factor; PGC1α, Peroxisome proliferator-activated receptor gamma coactivator 1-α; Hnf4ɑ; hepatocyte nuclear factor 4-α; GR, glucocorticoid receptor; FOXO-1, Forkhead box protein O1

### Luciferase reporter assay

The HEK-293 cells were plated in 24-well plates and co-transfected with 0.5 µg pcDNA3.1-MEF2C plasmid, 0.2 µg reporter plasmid, and 0.02 µg pRL-TK plasmid. After transfection for 48 h, the cell lysates were prepared, and reporter activities were evaluated using the Dual-Luciferase Reporter Assay System (Promega) according to the manufacturer’s instructions.

### Chromatin immunoprecipitation (ChIP) assays

For immunoprecipitation, 20 µL of protein A/G magnetic beads slurry (88802; Thermo Fisher Scientific) was washed with lysis buffer and incubated with 2 µg anti-MEF2C antibody (ab211493; Abcam, Cambridge, UK) or normal rabbit IgG antibody (CST) in 200 µL lysis buffer for 4 h at 4°C. The antibody-bound beads were washed three times with lysis buffer, and incubated with the supernatant of cell lysates overnight at 4°C. For DNA extraction, the beads were resuspended in 400 µL 100 mM Tris-HCl pH 7.5, 50 mM NaCl, 10 mM ethylenediaminetetraacetic acid containing 10 mg/mL protease K (Millipore) and incubated for 30 min at 37°C. Finally, the MEF2C-binding DNA was obtained using phenol-chloroform extraction. Extracted DNA sample (the input sample and ChIP DNA sample) was used for quantitative PCR amplification using AChR-β primers (forward, 5’-TACAGTCCTGAACCACTTGCC-3’; reverse, 5’-GCTAGGTGATATTGAAGAGC-3’).

### Statistical analysis

For pairwise comparison analysis, data are presented as mean ± standard error. Statistical significance was assessed using an unpaired Student’s t-test and a one- or two-way analysis of variance with Bonferroni post hoc comparisons using SPSS software (v.22.0; IBM Corp., Armonk, NY, USA). *P* < 0.05 was considered statistically significant.

## Results

### Increase in IL-6 levels during aging inhibited AChR-β expression in skeletal muscle

**Fig. 1:**
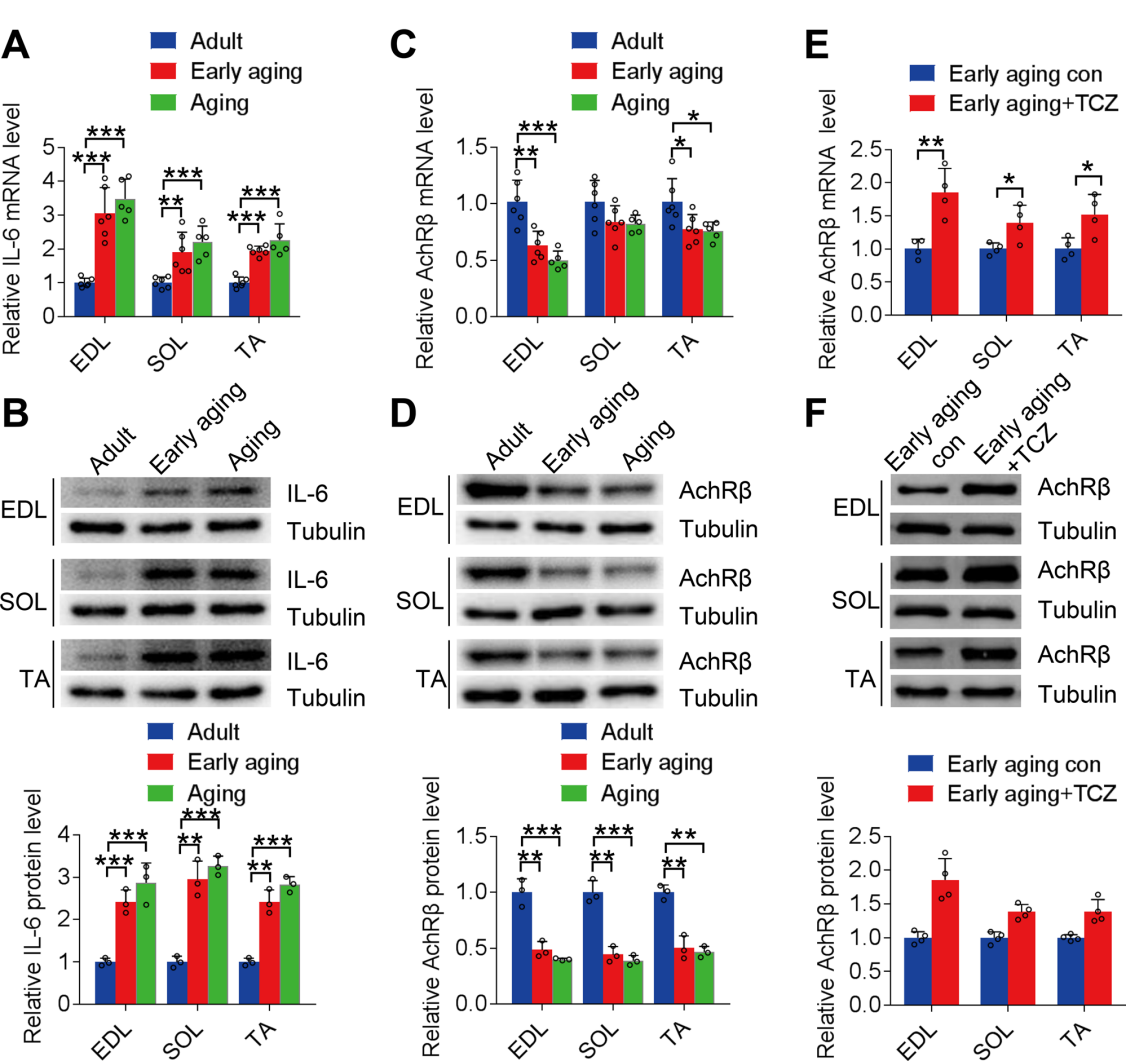
Acetylcholine receptor β-subunit (AChR-β) expression in skeletal muscle is inhibited by interleukin (IL)-6 during aging. **A** and **B**: IL-6 gene and protein expression levels in the extensor digitorum longus (EDL), gastrocnemius soleus (SOL), and tibialis anterior (TA) muscles during aging. **C** and **D**: AChR-β gene and protein expression levels in EDL, SOL, and TA muscles during aging. **E** and **F**: AChR-β gene and protein expression levels in early-aging mice after tocilizumab treatment. *P < 0.05, **P < 0.01, ***P < 0.001. Each group comprised three males and three females, *n* = 6. The data are from three independent experiments

First, serum and skeletal muscle IL-6 expression levels were analyzed in adult, early-aging, and aging mice. Compared with that of eight-week-old adult mice, plasma IL-6 levels of early-aging and aging mice were significantly elevated (*P* < 0.01; see Additional file Fig. S1). IL-6 gene and protein expression levels in all skeletal muscles increased significantly with aging (*P* < 0.01; Fig. 1A and B); EDL showed the most significant increase. Moreover, AChR-β gene expression and protein levels in all skeletal muscles of early-aging and aging mice were reduced compared with those in adult mice; the EDL (*P* < 0.01) and TA (*P* < 0.05) showed the most significant reductions (Fig. 1C and D).

Next, tocilizumab, the IL-6 receptor antibody, was used to block the action of IL-6 (Tanaka et al. 2018). Following the intraperitoneal injection of tocilizumab, serum IL-6 levels in early-aging mice remained unaffected (*P* = 0.08; see Additional file Fig. S1). AChR-β gene expression and protein levels in all skeletal muscles were significantly elevated (mRNA expression, + 1.85-fold vs. early aging con, *P* = 0.0049 in EDL, + 1.39-fold vs. early aging con, *P* = 0.0316 in SOL, + 1.98-fold vs. early aging con, *P* < 0.0039 in TA; protein expression, + 1.86-fold vs. early aging con, *P* = 0.0019 in EDL, + 1.38-fold vs. early aging con, *P* = 0.0015 in SOL, + 1.39-fold vs. early aging con, *P* = 0.0053 in TA), especially in the EDL (Fig. 1E and F).

### PGC1α regulated the effects of IL-6 on AChR-β expression in skeletal muscles

#### Inhibition of IL-6 reversed the decrease in PGC1α expression in fast-twitch skeletal muscle during aging

**Fig. 2:**
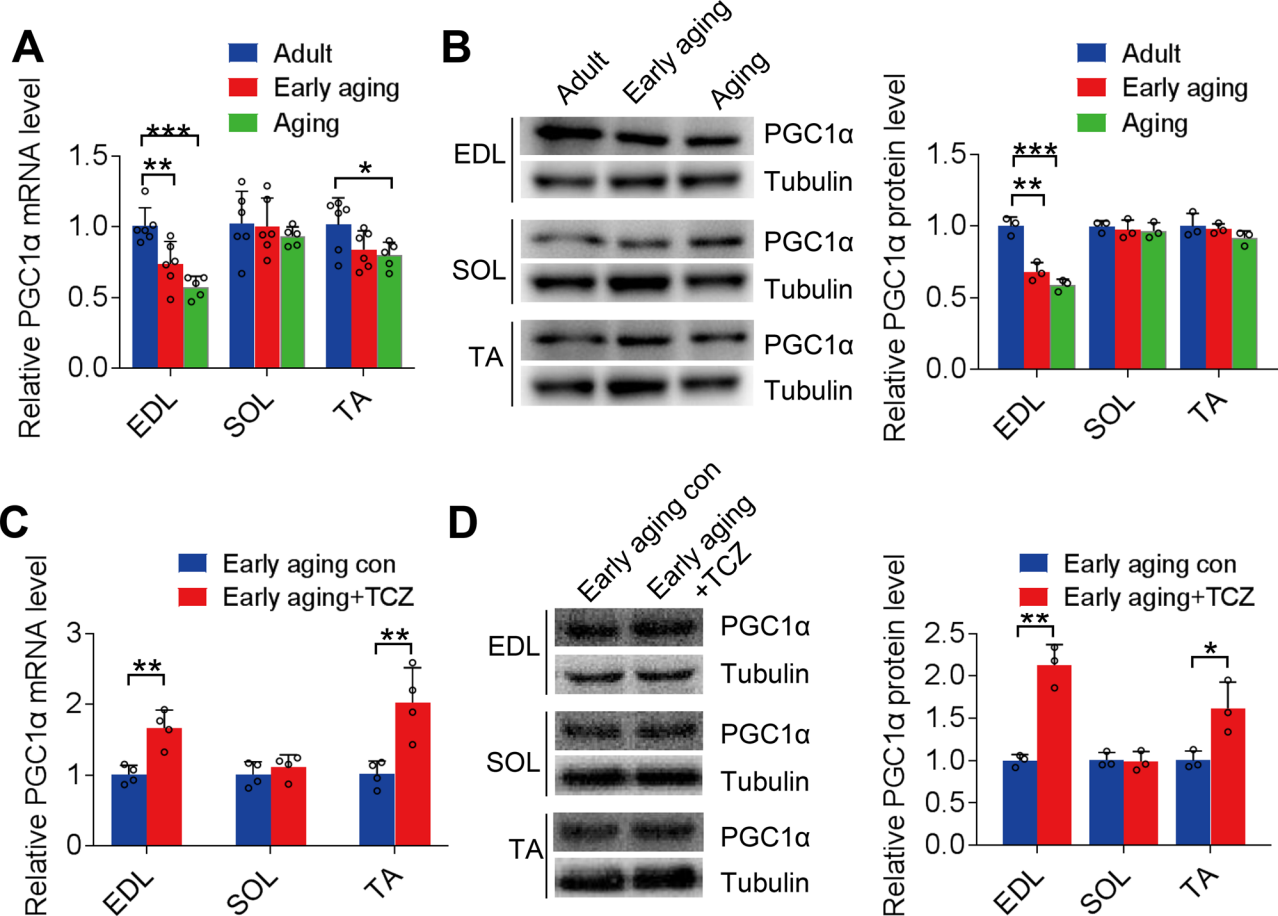
Interleukin (IL)-6-meditated peroxisome proliferator-activated receptor gamma coactivator 1-α (PGC1α) inhibition in skeletal muscle during aging. **A** and **B**: PGC1α gene and protein expression levels in EDL, SOL, and TA muscles during aging. **C** and **D**: PGC1α gene and protein expression levels in early-aging mice after treatment with tocilizumab. EDL, extensor digitorum longus; SOL, gastrocnemius soleus; TA, tibialis anterior. *P < 0.05, **P < 0.01, ***P < 0.001. Each group comprised three males and three females. *n* = 6. The data are from three independent experiments

The correlation between the decline in skeletal muscle contractile activity and mitochondrial function during aging may be linked to the transcription cofactor PGC1α (Gill et al. 2018). In the comparative analysis of PGC1α expression in the skeletal muscle of adult, early-aging, and aging mice, the gene and protein expression of PGC1α in the EDL and TA muscles decreased during aging, with EDL exhibiting the most significant decrease (*P* < 0.01; Fig. 2A and B). When tocilizumab was administered, the decrease in PGC1α expression in the EDL and TA muscles of the early-aging mice was reversed (*P* < 0.01; Fig. 2C and D).

#### PGC1α regulated the effects of IL-6 on AChR-β expression

**Fig. 3:**
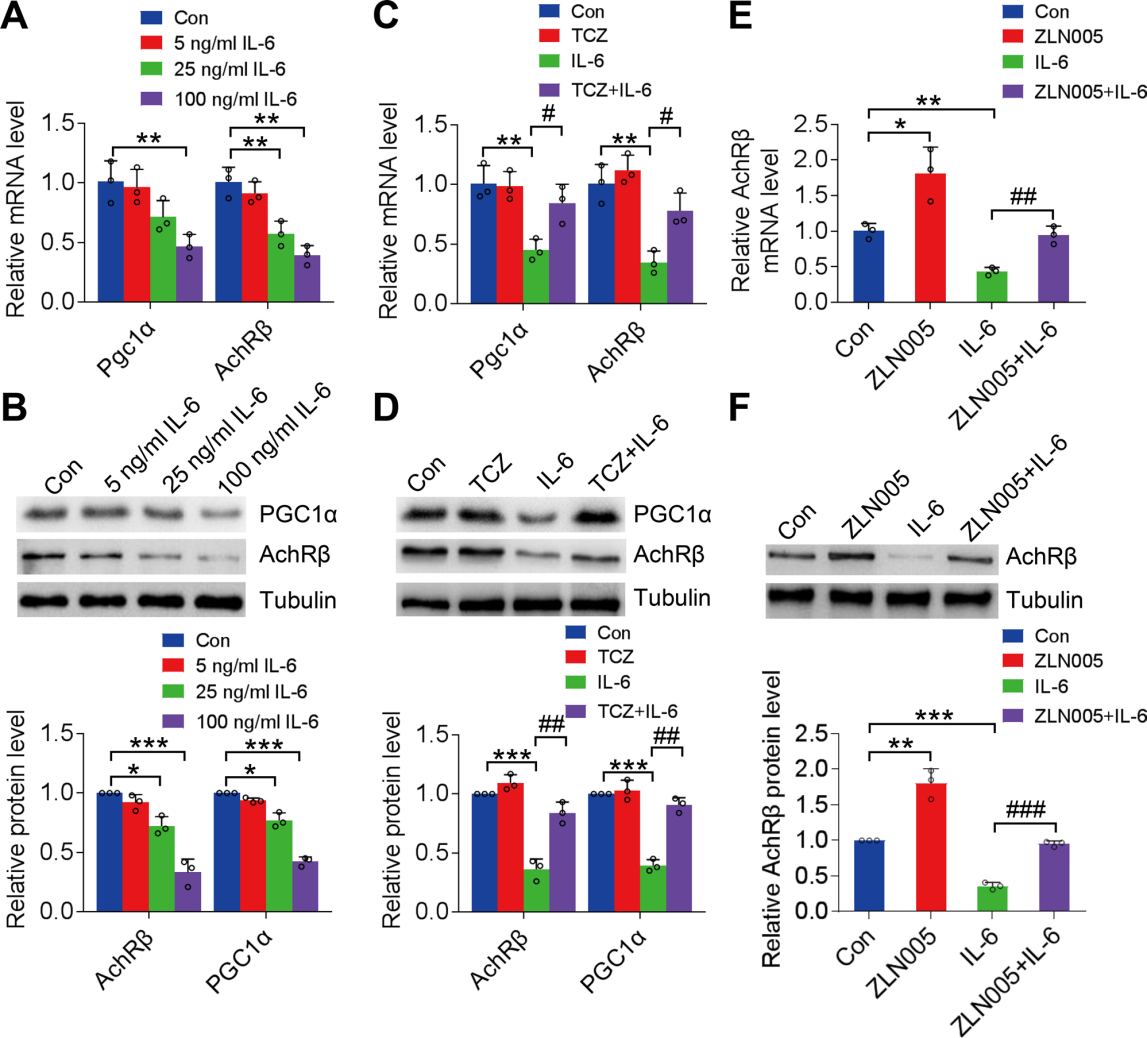
PGC1α regulates the inhibition of AChR-β expression by IL-6. **A**: PGC1α and AChR-β gene expression in C2C12 myotubes following treatment with 5, 25, and 100 ng/mL of IL-6. **B**: PGC1α and AChR-β protein expression levels in C2C12 myotubes after treatment with IL-6. **C**: PGC1α and AChR-β gene expression in IL-6-treated C2C12 myotubes after treatment with 5 µg/mL tocilizumab. **D**: PGC1α and AChR-β protein expression levels in IL-6-treated (100 ng/mL) C2C12 myotubes after treatment with tocilizumab. **E** and **F**: AChR-β gene and protein expression levels in IL-6-treated C2C12 myotubes after treatment with ZLN005. PGC1α, peroxisome proliferator-activated receptor gamma coactivator 1-α; AChR-β, acetylcholine receptor β-subunit; IL, interleukin. *n* = 3. *P < 0.05, **P < 0.01, ***P < 0.001, #*P* < 0.05, ##*P* < 0.01, ###*P* < 0.001. The data are from three independent experiments

IL-6 (5, 25, and 100 ng/mL) was applied to C2C12 myotubes [see Additional file Fig. S2], for 24 h to model long-term IL-6 treatment (Muñoz-Cánoves et al. 2013). The results showed that IL-6 inhibited AChR-β and PGC1α gene expressions in a dose-dependent manner (Fig. 3A). Simultaneously, cytoplasmic AChR-β and PGC1α protein levels gradually decreased with the increase in IL-6 concentration (Fig. 3B). Pretreatment of skeletal muscle cells with tocilizumab (5 µg/mL) reversed the inhibitory effect of IL-6 (100 ng/mL) on AChR-β and PGC1α gene (*P* < 0.05; Fig. 3C) and protein (*P* < 0.01; Fig. 3D) expression levels.

Additionally, the administration of ZLN005 (10 µM, a PGC1α agonist) promoted baseline AChR-β expression in skeletal muscle cells (*P* < 0.001); in contrast, 10 µM of SR18292, a PGC1α inhibitor, significantly inhibited the AChR-β expression (*P* < 0.01) [see Additional file Fig. S3]. In C2C12 myotubes treated with 100 ng/mL of IL-6, the addition of ZLN005 reversed the IL-6-mediated inhibition of AChR-β gene expression (+ 2.17-fold vs. IL-6, *P* = 0.0029) and protein expression (+ 2.65-fold vs. IL-6, *P* = 0.0001) (Fig. 3E and F). These results suggest that PGC1α regulates the inhibition of AChR-β expression by IL-6.

### ERK1/2 was the key cytoplasmic factor involved in the regulation of AChR-β expression by IL-6

**Fig. 4:**
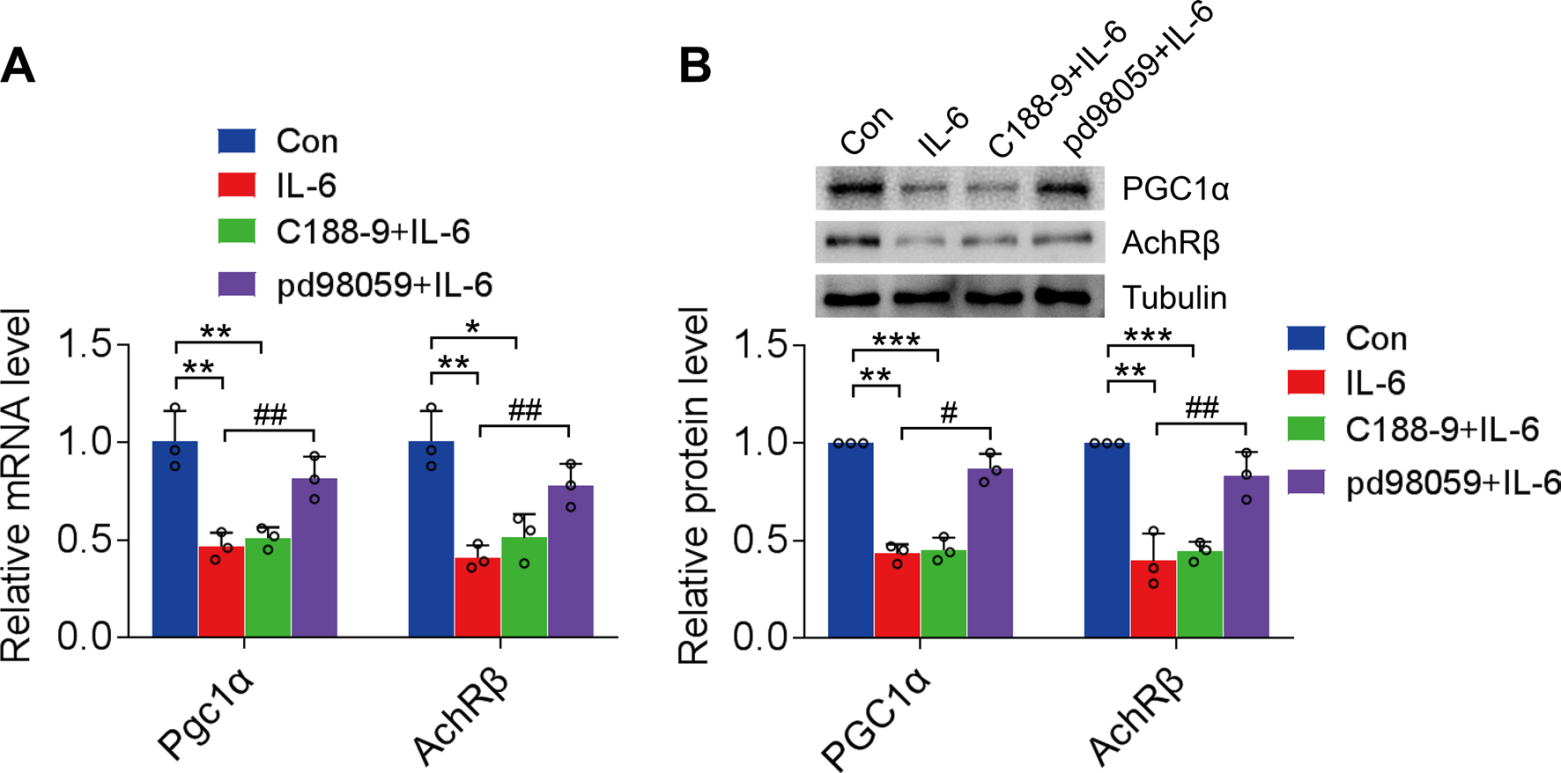
ERK1/2 activates PGC1α-mediated regulation of AChR-β expression following IL-6 treatment. **A**: PGC1α and AChR-β gene expression in IL-6-treated C2C12 myotubes following administration of STAT3 and ERK1/2 inhibitors, C188-9 (10 µM) and pd98059 (20 µM), respectively. **B**: PGC1α and AChR-β protein expression levels in IL-6-treated C2C12 myotubes following administration of STAT3 and ERK1/2 inhibitors. ERK 1/2, extracellular signal-regulated kinase; PGC1α, peroxisome proliferator-activated receptor gamma coactivator 1-α; AChR-β, acetylcholine receptor β-subunit; IL, interleukin; STAT3, signal transducer and activator of transcription 3. *n* = 3. *P < 0.05, **P < 0.01, ***P < 0.001, #*P* < 0.05, ##*P* < 0.01. The data are from three independent experiments

The cytoplasmic IL-6/IL-6R signaling pathway involved in the regulation of AChR-β was investigated by administering STAT3 inhibitor C188-9 (HY-112288, MCE; 10 µM) (Silva et al. 2015) and ERK1/2 inhibitor pd98059 (HY-12028, MCE; 20 µM) in C2C12 myotubes (Paola et al. 2010). C188-9 did not reverse the inhibitory effect of IL-6 on PGC1α and AChR-β (*P* > 0.05), whereas pd98059 significantly enhanced PGC1α (mRNA expression, vs. IL-6, *P* < 0.01; protein expression, vs. IL-6, *P* < 0.05,) and AChR-β (mRNA expression, vs. IL-6, *P* < 0.01; protein expression, vs. IL-6, *P* < 0.01) expression in C2C12 myotubes following treatment with IL-6, thereby restoring the expression to baseline levels (Fig. 4A and B).

### Transcription factor MEF2C was the key transcription factor recruited by PGC1α to regulate AChR-β expression

#### Combination of PGC1α and MEF2C mediated the regulation of AChR-β expression in skeletal muscles

PGC1α has been theorized to recruit other transcription factors and form a complex to regulate the transcription of target genes (Chen et al. 2022). Eight transcription factors, MEF2C/delta8 [T01769], MEF2DAB [T02505], estrogen-related receptor 1 [T04849], peroxisome proliferator-activated receptor (PPAR)-gamma: RXR-alpha [T05236], PPARα [T00694], glucocorticoid receptor [T00335], hepatocyte nuclear factor 4-α [T05287], and forkhead box protein O1 [T04203], were selected based on the bioinformatics analysis using the PROMO database, which predicted binding sites with the AChR-β promoter and also interacting with PGC1α.

**Fig. 5:**
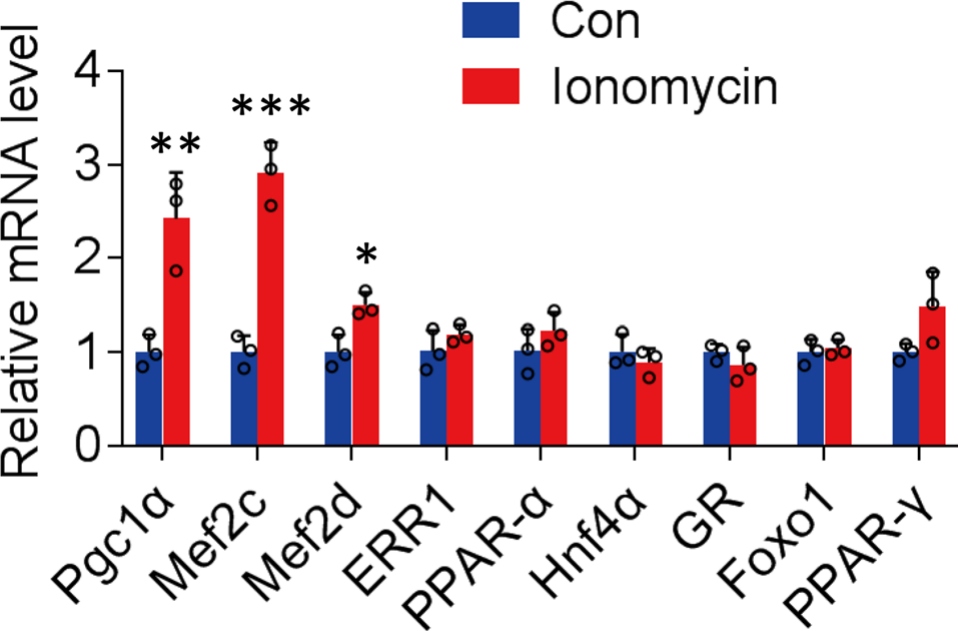
MEF2C was selected as the candidate transcription factor recruited by PGC1α. The expression of PGC1α and the eight candidate transcription factors in C2C12 myotubes induced by ionomycin was determined based on the bioinformatics analysis using the PROMO database. *n* = 3. *P < 0.05. **P < 0.01. ***P < 0.001

**Fig. 6:**
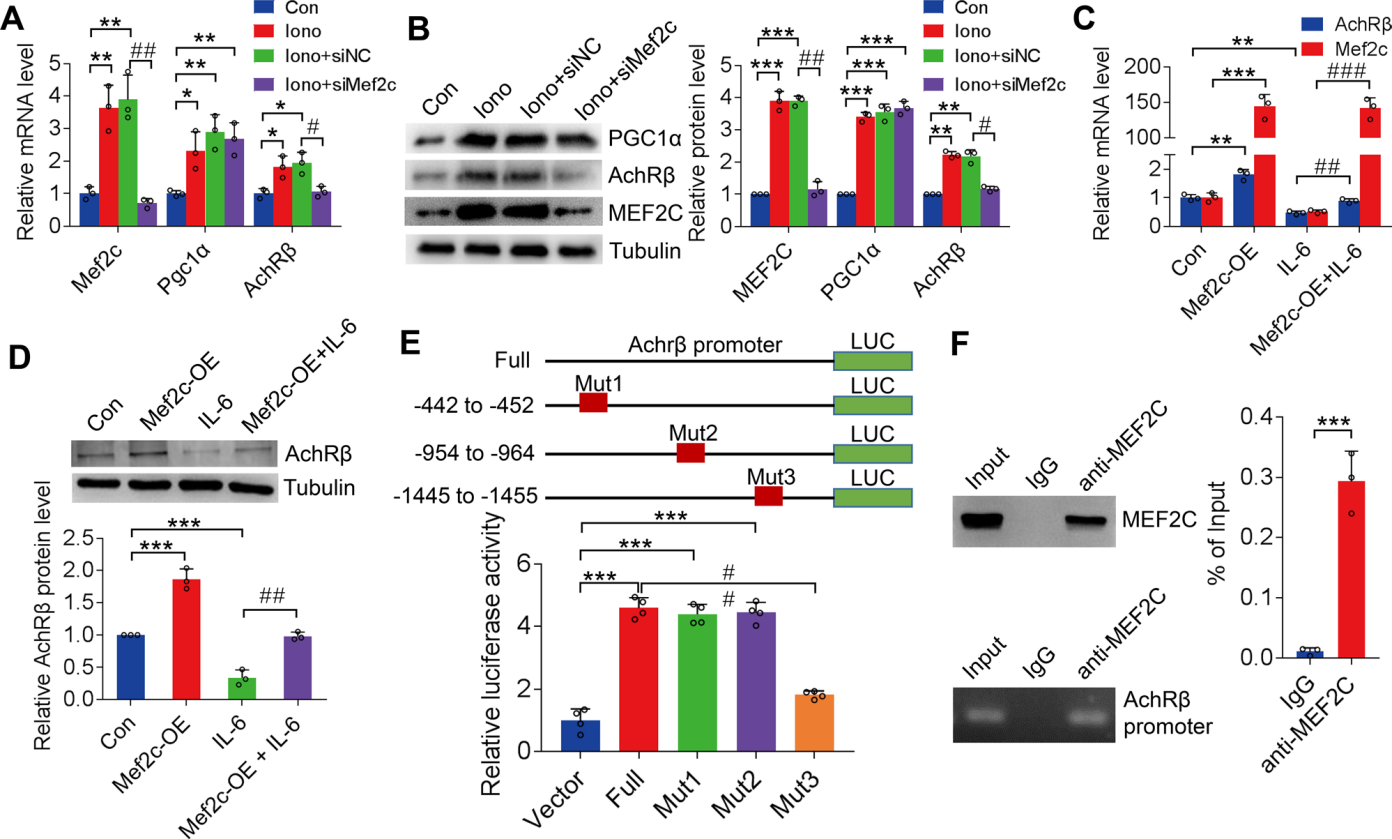
MEF2C recruitment by PGC1α targeting AChR-β promoter sequences to regulate transcription in skeletal muscle. **A** and **B**: PGC1α and AChR-β gene and protein expression in ionomycin-treated (0.5 µM) C2C12 myotubes following treatment with MEF2C-RNAi. **C** and **D**: AChR-β gene and protein expression levels in IL-6-treated C2C12 myotubes following MEF2C overexpression. **E**: Three binding sites, − 442 to − 452, −954 to − 964, and − 1445 to − 1455, were predicted by bioinformatics analysis using the PROMO database. **F**: Accordingly, − 1445 to − 1455, was determined to have the highest dural luciferase activity compared to other regions and was, thus, set as possible binding sites. **G**: ChIP assays demonstrated that MEF2C bound at − 1445 to − 1455 in the promoter region. MEF2C, myocyte enhancer factor 2 C; PGC1α, peroxisome proliferator-activated receptor gamma coactivator 1-α; AChR-β, acetylcholine receptor β-subunit; IL, interleukin; ChIP, chromatin immunoprecipitation.*P < 0.05, **P < 0.01, ***P < 0.001, #*P* < 0.05, ##*P* < 0.01, ###*P* < 0.001. The data are from three independent experiments

MEF2C was selected as the candidate transcription factor recruited by PGC1α [Fig. 5] based on the coordinative increase in PGC1α and MEF2C levels in C2C12 myotubes induced by increased intramyocellular calcium levels following treatment with ionomycin (HY-13434, MCE; 0.5 µM) (Huang et al. 2019). Further, the MEF2C-RNAi was constructed [see Additional file Fig. S4], which significantly reduced ionomycin-induced AChR-β expression (*P* < 0.05; Fig. 6A and B). MEF2C overexpression [see Additional file Fig. S4] significantly promoted AChRβ expression in myotubes and reversed the inhibition of AChR-β expression by IL-6 (*P* < 0.01; Fig. 6C and D).

#### Interactions between MEF2C and the AChR-β promoter

Three binding sites, − 442 to − 452; − 954 to − 964; and − 1445 to − 1455, were predicted through bioinformatics analysis using the PROMO database for investigating the interactions between MEF2C and the *AChR-β* promoter [see Additional file Fig. S5]. A full-length luciferase reporter gene plasmid encoding the wild-type (WT) *AChR-β* promoter region, three mutant plasmids, and transcription factor plasmids were constructed (Fig. 6E). The luciferase reporter gene WT plasmid, mutant plasmid, and transcription factor plasmid were co-transfected into HEK-293 cells, and the resulting fluorescence intensity was examined. Fluorescence ratio values were significantly higher than those in other sites (*P* < 0.01; Fig. 6E), indicating that this site constitutes the binding site for MEF2C in the AChR-β promoter region. Furthermore, the MEF2C binding of the − 1445 to − 1455 region was verified using qPCR-ChIP analyses following ionomycin stimulation of C2C12 cells (*P* < 0.001; Fig. 6F).

#### MEF2C decreased during aging and was affected by IL-6

**Fig. 7:**
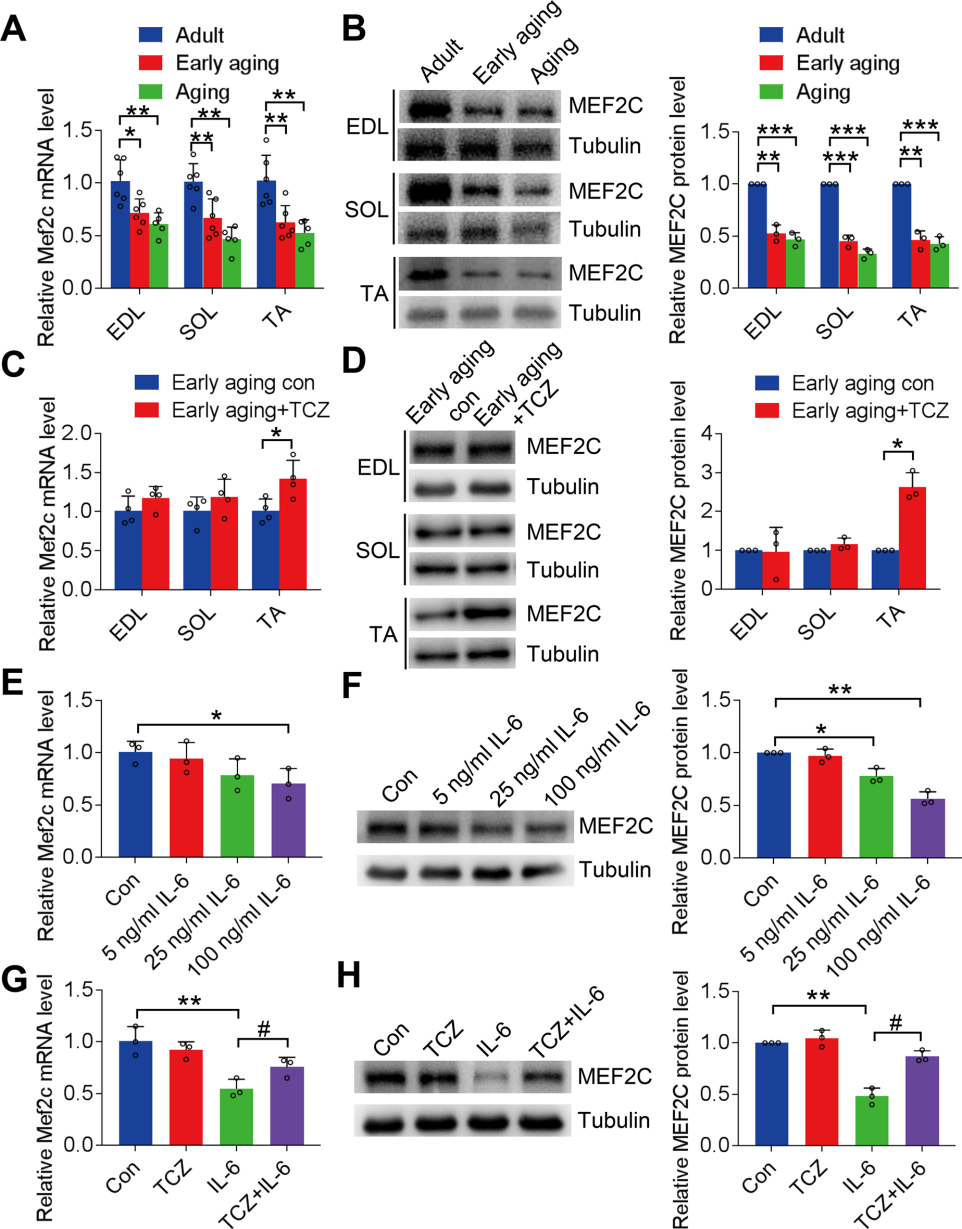
Myocyte enhancer factor 2 C (MEF2C) expression in skeletal muscle was inhibited by interleukin (IL)-6. **A** and **B**: MEF2C expression in EDL, SOL, and TA muscles during aging. **C** and **D**: MEF2C expression in early-aging mice after treatment with tocilizumab. **E** and **F**: MEF2C gene and protein expression in C2C12 myotubes following treatment with 5, 25, and 100 ng/mL of IL-6. **G** and **H**: MEF2C gene and protein expression in IL-6-treated C2C12 myotubes with tocilizumab. EDL, extensor digitorum longus; SOL, gastrocnemius soleus; TA, tibialis anterior. *P < 0.05. **P < 0.01, ***P < 0.001. #*P* < 0.05. In vivo, *n* = 6; in vitro, *n* = 3. The data are from three independent experiments

MEF2C expression in the EDL, SOL, and TA in early-aging and aging mice was significantly lower than that in eight-week-old adult mice (Fig. 7A and B), and this trend intensified with aging. When tocilizumab was administered, the decrease in MEF2C expression in skeletal muscles of early-aging mice was reversed in the TA (*P* < 0.05), whereas no significant changes were observed in the EDL and SOL (*P* > 0.05; Fig. 7C and D).

Further, the administration of different concentrations of IL-6 (5, 25, and 100 ng/mL) in C2C12 myotubes for 24 h resulted in a gradual decrease in MEF2C expression, indicated by an increase in IL-6 concentration (Fig. 7E and F). In addition, the inhibitory action of IL-6 (100 ng/mL) on MEF2C gene (*P* < 0.05; Fig. 7G) and protein (*P* < 0.05; Fig. 7H) expression was reversed by pretreatment with tocilizumab. Therefore, both in vitro and in vivo studies show that IL-6 inhibited MEF2C expression.

## Discussion

LGI is a major causal factor that promotes aging. Considering that myokines are secreted by skeletal muscle, it is plausible that there is an autocrine effect on the muscle itself. To our knowledge, the present study is the first to report that IL-6 expression is gradually increased in mouse skeletal muscle during aging. Moreover, the study is the first to report that IL-6 binds to IL-6R on the skeletal muscle surface to inhibit AChR-β expression through the ERK1/2-PGC1/MEF2C signaling pathway, resulting in NMJ dysfunction and a decline in muscle contraction. Thus, skeletal muscle may actively change its contraction to adapt to aging through the autocrine effect of myokines.

Skeletal muscle atrophy and strength loss are typical features of sarcopenia. Patients with sarcopenia consistently display a severe decline in motor function upon reaching a stage where effective clinical intervention is challenging (Correa-de-Araujo and Bhasin 2022). Therefore, it is necessary to explore and identify the changes in skeletal muscle in the early stages of aging, i.e., the pre-sarcopenia stage. The stage is characterized by the decline in contraction without typical structural changes, such as muscle atrophy, and the decrease in new muscle fibers (Schaap et al. 2013, 2018). Muscle contractions are triggered by acetylcholine signal transduction at the NMJ. which relies on AChRs. The AChRs are heteromeric transmembrane proteins composed of four constitutive subunits (2α, 1β, and 1δ), and one variable γ subunit, which switches to ε during muscle development to form adult AChRs, on the postsynaptic membrane. After the development period, the NMJs of adult mice maintain stability for a long time; however, once mice reach 12–18 months of age (early aging), a small proportion of their NMJs show a loss of motor terminal branches and AChRs (Jang and Remmen 2011). With advancing age, AChRs are further decreased, and increased endplate fragmentation and reduced overlapping between the nerve terminal and AChRs contribute to NMJ dysfunction (Bao et al. 2020). This study demonstrates that AChR-β expression in the skeletal muscle decreases in early-aging mice. Considering the necessity of AChR-β for NMJ function (Papke and Lindstrom 2020), the decline in AChR expression, which represents muscle dysfunction, is possibly a salient manifestation of pre-sarcopenia. Therefore, future studies must focus on the changes in the AChRs-NMJ in skeletal muscles and develop novel methods and standards to detect these pre-sarcopenia changes.

During aging, muscle atrophy, increased fiber heterogeneity, and decreased muscle fibers occur, especially for fast-twitch (type II) fibers (Nishikawa et al. 2021). As type II fibers can produce a higher maximal contractile force than that produced by slow-twitch fibers, selective loss of those fibers causes muscle strength loss in adults over 55 years of age (Verdijk et al. 2010). Our study indicated that AChR-β expression in both fast-twitch and slow-twitch muscles decreased with aging, with the most significant decrease occurring in the fast EDL, representing the possible principal molecular mechanisms of pre-sarcopenia. Therefore, AChR imaging and quantitative analysis may be potential biomarkers to assess skeletal muscle function during aging (Hu et al. 2021).

Aging-related LGI is characterized by high levels of circulating pro-inflammatory markers, such as IL-6, primarily secreted by active skeletal muscle cells (Pedersen and Fischer 2007). During aging, IL-6 contributes to decreased skeletal muscle strength, mass, function, and training-mediated adaptation (Grosicki et al. 2020; Visser et al. 2002). Population data have indicated that IL-6 and tumor necrosis factor α levels are significantly increased in older adults with sarcopenia (Bian et al. 2017); the higher the levels of IL-6 and C-reactive protein, the greater the risk of muscle loss (Schaap et al. 2006). In transgenic mice with IL-6 overexpression, muscle atrophy can be reversed entirely following treatment with an IL-6 receptor antibody (Tsujinaka et al. 1996). Long-term exposure to high IL-6 levels can result in muscle atrophy and muscle strength decline by promoting muscle catabolism (Pelosi et al. 2021). In 2020, a randomized controlled trial concluded that the age-related increase in circulating IL-6 levels was a key factor in the decline in skeletal muscle strength, mass, function, and training-mediated adaptations among 99 older adults (Grosicki et al. 2020). In this study, serum and skeletal muscle IL-6 levels in mice gradually increased with aging, whereas skeletal muscle AChR-β expression gradually declined; the AChR-β expression in skeletal muscle was significantly increased with the administration of IL-6R antibody. As demonstrated in aging C2C12 myoblasts induced by C2-ceramide at a concentration of 50 mM for 8 h, the IL-6 gene and protein expression increased, whereas that of PGC1α, MEF2C, and AChR-β decreased (see Additional file Fig. S6). Thus, the chronic inflammation induced by IL-6 with aging may be a major reason for the changes in AChR-β in skeletal muscle. IL-6 secretion is an active change in aging skeletal muscle and may be a promising target for improving skeletal muscle strength (Yano et al. 2019; Pototschnig et al. 2023).

PGC1α is a key factor in regulating mitochondrial function and skeletal muscle metabolism and plays a major role in the differentiation and maintenance of skeletal muscle fibers (Halling et al. 2019). Our study, in parallel with previous reports (Gill et al. 2018), found that PGC1α levels gradually declined with aging. Furthermore, the decline in PGC1α was inversely correlated with the increase in IL-6 secretion during aging, and the IL-6 receptor antibody significantly increased both PGC1α and AChR-β expression. The activation of PGC1α reverses the inhibitory effect of IL-6 on AChR-β expression, consistent with a previous study reporting that the overexpression of PGC1α improves AChR expression (Handschin et al. 2007). Thus, activation of the PGC1α pathway promotes the upregulation of AChR-β expression and stabilizes the NMJ (Arnold et al. 2014).

PGC1α expression is regulated by the activation of reactive oxygen species through the ERK1/2-cyclic adenosine triphosphate response element-binding protein pathway (Espinoza et al. 2017). Consistent with this notion, our study revealed that the IL-6/IL-6R autocrine effect of skeletal muscles regulates the expression of PGC1α by the ERK1/2 pathway instead of the classic inflammatory STAT3 pathway, which further enters the nucleus to influence the expression of AChR-β. As a transcription cofactor, PGC1α often forms complexes with other transcription factors to mediate the regulation of target gene expression. Based on the bioinformatics prediction, as verified by dual-luciferase reporter gene technology, our results showed that MEF2C directly binds to the promoter of AChR-β. Consistent with the age-related changes in MEF2C reported previously (Taylor and Hughes 2017), the MEF2C levels also decreased in early-aging mice in the present study, although IL-6 receptor antibodies could not reverse this decline. Hence, activation of the ERK1/2-PGC1α signaling pathway recruits MEF2C to regulate AChR-β expression. As a member of the MEF2 family, MEF2C plays an essential role in skeletal muscle growth, proliferation, differentiation, and the maintenance of fiber type (Dong et al. 2017; Potthoff et al. 2007). The results of this study demonstrate that PGC1α/MEF2C may be significant in regulating AChR-β expression throughout the growth cycle of the body.

**Fig. 8:**
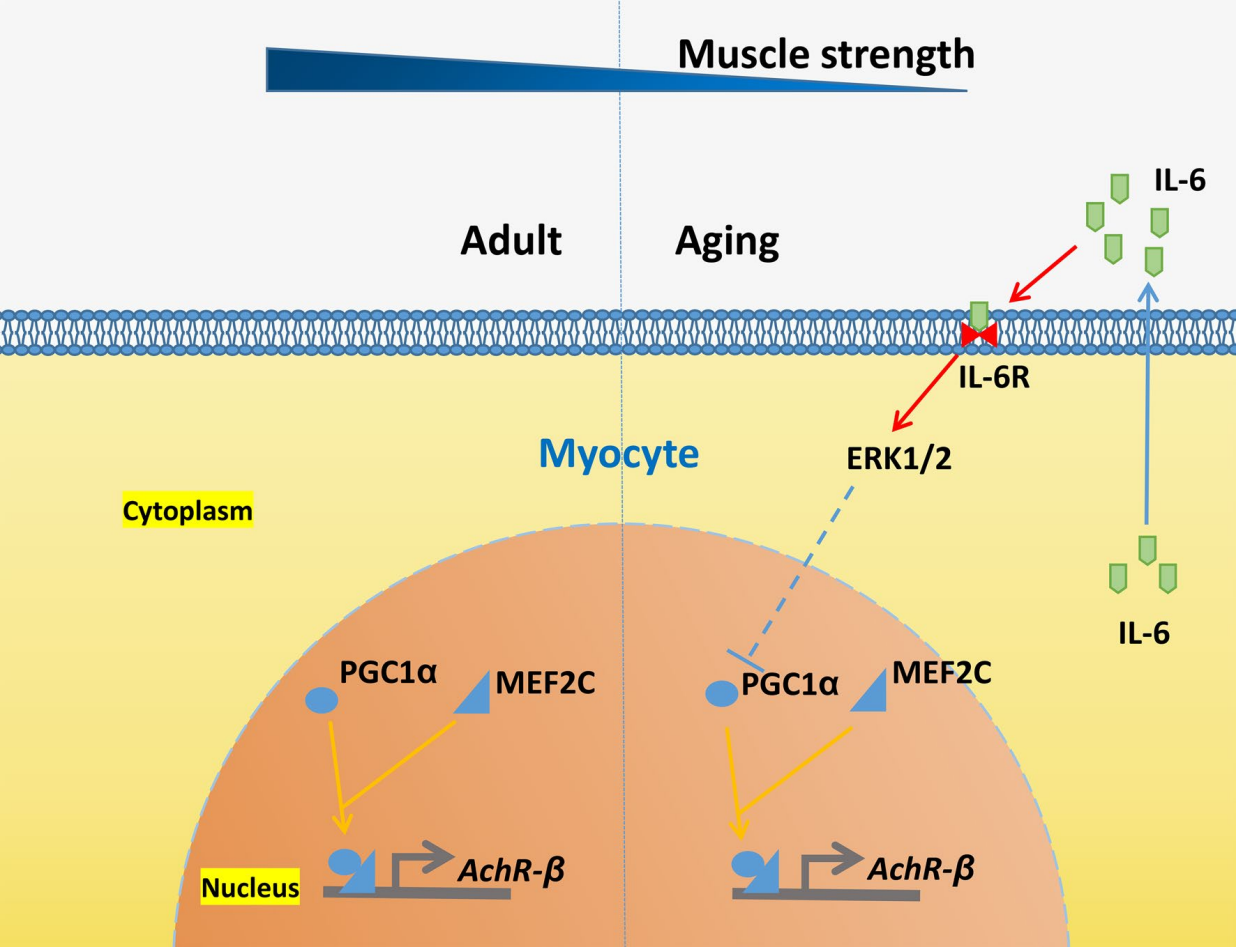
Schematic representation of the study results. IL-6, interleukin-6; IL-6R, IL-6 receptor; ERK 1/2, extracellular signal-regulated kinase; PGC1α, peroxisome proliferator-activated receptor gamma coactivator 1-α; MEF2C, myocyte enhancer factor 2 C; AChR-β, acetylcholine receptor β-subunit

The present study is the first to describe the autocrine effect of IL-6 on skeletal muscle. The autocrine effects of IL-6, which differs from those of classic inflammatory effects, directly regulate AChR-β expression and skeletal muscle contraction by recruiting MEF2C through the ERK1/2-PGC1α pathway. As reported by Yang et al. (Yang et al. 2022), the function of PGC1α and the mitochondria of skeletal muscle can also be inhibited by IL-6. Therefore, our data are the first to verify the effect by which the IL-6-ERK1/2-PGC1α/MEF2C signaling pathway regulates AChR-β expression and NMJ function (Fig. 8). Furthermore, the study demonstrates a broad and complex interaction between chronic inflammation, exercise capacity, mitochondrial function, and energy metabolism in aging skeletal muscle (Fealy et al. 2021; Gill et al. 2019).

## Conclusions

This study verified the key role of myokines, specifically IL-6, in skeletal muscle aging, thereby suggesting the necessity to reevaluate the role of skeletal muscles during aging beyond the passive changes following motoneuron degeneration. In the future, the regulation of AChR-β by the IL-6/IL-6R-ERK1/2-PGC1α/MEF2C pathway should be investigated in patients and animals with sarcopenia. This will enable exploration of the potential of myokine-autocrine interactions in predicting and developing new strategies to monitor, maintain, and improve skeletal muscle structure and function.

## Electronic supplementary material

Below is the link to the electronic supplementary material.

Supplementary Material 1

## Data Availability

No datasets were generated or analysed during the current study.

## Abbreviations

AChR: acetylcholine receptor
AChR-β: AChR β-subunit
DMEM: Dulbecco’s modified Eagle medium
EDL: extensor digitorum longus
ERK1/2: extracellular signal-regulated kinases 1/2, IL, interleukin
LGI: low-grade inflammation
MEF2C: myocyte enhancer factor 2C
NMJ: neuromuscular junction
PBS: phosphate-buffered saline
PGC1α: peroxisome proliferator-activated receptor gamma coactivator 1-α
SOL: gastrocnemius soleus
STAT3: signal transducer and activator of transcription 3
TA: tibialis anterior
cDNA: complementary DNA
pcDNA: plasmid cloning DNA
siRNA: small interfering RNA
PCR: polymerase chain reaction
ChIP: chromatin immunoprecipitation
PPAR: peroxisome proliferator-activated receptor

## References

[CR11] Alchin DR. Sarcopenia: describing rather than defining a condition. J Cachexia Sarcopenia Muscle. 2014;5:265–8. 10.1007/s13539-014-0156-8. 25092476 10.1007/s13539-014-0156-8 PMC4248413

[CR53] Arnold AS, Gill J, Christe M, Ruiz R, McGuirk S, St-Pierre J, et al. Morphological and functional remodelling of the neuromuscular junction by skeletal muscle PGC-1α. Nat Commun. 2014;5:3569. 10.1038/ncomms4569. 24686533 10.1038/ncomms4569 PMC4846352

[CR37] Bao Z, Cui C, Chow SK, Qin L, Wong RMY, Cheung WH. AChRs degeneration at NMJ in aging-associated sarcopenia-a systematic review. Front Aging Neurosci. 2020;12:597811. 10.3389/fnagi.2020.597811. 33362532 10.3389/fnagi.2020.597811 PMC7759742

[CR45] Bian AL, Hu HY, Rong YD, Wang J, Wang JX, Zhou XZ. A study on relationship between elderly Sarcopenia and inflammatory factors IL-6 and TNF-α. Eur J Med Res. 2017;22:25. 10.1186/s40001-017-0266-9. 28701179 10.1186/s40001-017-0266-9 PMC5508730

[CR15] Blasco A, Gras S, Mòdol-Caballero G, Tarabal O, Casanovas A, Piedrafita L, et al. Motoneuron deafferentation and gliosis occur in association with neuromuscular regressive changes during ageing in mice. J Cachexia Sarcopenia Muscle. 2020;11:1628–60. 10.1002/jcsm.12599. 32691534 10.1002/jcsm.12599 PMC7749545

[CR32] Chen L, Qin Y, Liu B, Gao M, Li A, Li X, et al. PGC-1α-mediated mitochondrial quality control: molecular mechanisms and implications for heart failure. Front Cell Dev Biol. 2022;10:871357. 10.3389/fcell.2022.871357. 35721484 10.3389/fcell.2022.871357 PMC9199988

[CR33] Correa-de-Araujo R, Bhasin S. Public health need, molecular targets, and opportunities for the accelerated development of function-promoting therapies: Proceedings of a National Institute on Aging Workshop. Proceedings of a National institute on aging workshop. J Gerontol A Biol Sci Med Sci. 2022;77:2227–37. 10.1093/gerona/glac181 10.1093/gerona/glac181 PMC10148729 36399442

[CR1] Cruz-Jentoft AJ, Sayer AA, Sarcopenia. Lancet. 2019a;393:2636–46. 10.1016/S0140-6736(19)31138-9. 31171417 10.1016/S0140-6736(19)31138-9

[CR5] Cruz-Jentoft AJ, Bahat G, Bauer J, Boirie Y, Bruyère O, Cederholm T, et al. Sarcopenia: revised European consensus on definition and diagnosis. Age Ageing. 2019b;48:16–31. 10.1093/ageing/afy169. 30312372 10.1093/ageing/afy169 PMC6322506

[CR14] Darabid H, Perez-Gonzalez AP, Robitaille R. Neuromuscular synaptogenesis: coordinating partners with multiple functions. Nat Rev Neurosci. 2014;15:703–18. 0.1038/nrn3821. 25493308

[CR31] Di Paola R, Galuppo M, Mazzon E, Paterniti I, Bramanti P, Cuzzocrea S. PD98059, a specific MAP kinase inhibitor, attenuates multiple organ dysfunction syndrome/failure (MODS) induced by zymosan in mice. Pharmacol Res. 2010;61:175–87. 10.1016/j.phrs.2009.09.008. 19819333 10.1016/j.phrs.2009.09.008

[CR56] Dong C, Yang XZ, Zhang CY, Liu YY, Zhou RB, Cheng QD, et al. Myocyte enhancer factor 2 C and its directly-interacting proteins: a review. Prog Biophys Mol Biol. 2017;126:22–30. 10.1016/j.pbiomolbio.2017.02.002. 28163053 10.1016/j.pbiomolbio.2017.02.002

[CR54] Espinoza MB, Aedo JE, Zuloaga R, Valenzuela C, Molina A, Valdés JA. Cortisol induces reactive oxygen species through a membrane glucocorticoid receptor in rainbow trout myotubes. J Cell Biochem. 2017;118:718–25. 10.1002/jcb.25676. 27564718 10.1002/jcb.25676

[CR59] Fealy CE, Grevendonk L, Hoeks J, Hesselink MKC. Skeletal muscle mitochondrial network dynamics in metabolic disorders and aging. Trends Mol Med. 2021;27:1033–44. 10.1016/j.molmed.2021.07.013. 34417125 10.1016/j.molmed.2021.07.013

[CR8] Ferraro E, Molinari F, Berghella L. Molecular control of neuromuscular junction development. J Cachexia Sarcopenia Muscle. 2012;3:13–23. doi: 0.1007/s13539-011-0041-7. 10.1007/s13539-011-0041-7 PMC3302983 22450265

[CR3] Ferrucci L, de Cabo R, Knuth ND, Studenski S. Of Greek heroes, wiggling worms, mighty mice, and old body builders. J Gerontol Biol Sci Med Sci. 2012;67A:13–6. 10.1093/gerona/glr046. 10.1093/gerona/glr046 PMC3260484 22113943

[CR19] Forcina L, Franceschi C, Musarò A. The hormetic and hermetic role of IL-6. Ageing Res Rev. 2022;80:101697. 10.1016/j.arr.2022.101697. 35850167 10.1016/j.arr.2022.101697

[CR18] Fu XQ, Peng J, Wang AH, Luo ZG. Tumor necrosis factor alpha mediates neuromuscular synapse elimination. Cell Discov. 2020;6:9. 10.1038/s41421-020-0143-5. 32140252 10.1038/s41421-020-0143-5 PMC7051980

[CR16] Fulop T, Witkowski JM, Olivieri F, Larbi A. The integration of inflammaging in age-related diseases. Semin Immunol. 2018;40:17–35. 10.1016/j.smim.2018.09.003. 30287177 10.1016/j.smim.2018.09.003

[CR28] Gill JF, Santos G, Schnyder S, Handschin C. PGC-1α affects aging-related changes in muscle and motor function by modulating specific exercise-mediated changes in old mice. Aging Cell. 2018;17:e12697. 10.1111/acel.12697. 29067788 10.1111/acel.12697 PMC5770876

[CR60] Gill JF, Delezie J, Santos G, McGuirk S, Schnyder S, Frank S, et al. Peroxisome proliferator-activated receptor γ coactivator 1α regulates mitochondrial calcium homeostasis, sarcoplasmic reticulum stress, and cell death to mitigate skeletal muscle aging. Aging Cell. 2019;18:e12993. 10.1111/acel.12993. 31290266 10.1111/acel.12993 PMC6718523

[CR4] Goodpaster BH, Park SW, Harris TB, Kritchevsky SB, Nevitt M, Schwartz AV, et al. The loss of skeletal muscle strength, mass, and quality in older adults: the health, aging and body composition study. J Gerontol Biol Sci Med Sci. 2006;61:1059–64. 10.1093/gerona/61.10.1059. 10.1093/gerona/61.10.1059 17077199

[CR43] Grosicki GJ, Barrett BB, Englund DA, Liu C, Travison TG, Cederholm T, et al. Circulating interleukin-6 is associated with skeletal muscle strength, quality, and functional adaptation with exercise training in mobility-limited older adults. J Frailty Aging. 2020;9:57–63. 10.14283/jfa.2019.30.32150215 10.14283/jfa.2019.30

[CR6] Gustafsson T, Ulfhake B. Sarcopenia: what is the origin of this aging-induced disorder? Front Genet. 2021;12:688526. 10.3389/fgene.2021.688526. 34276788 10.3389/fgene.2021.688526 PMC8285098

[CR51] Halling JF, Jessen H, Nøhr-Meldgaard J, Buch BT, Christensen NM, Gudiksen A, et al. PGC-1α regulates mitochondrial properties beyond biogenesis with aging and exercise training. Am J Physiol Endocrinol Metab. 2019;317:E513–25. 10.1152/ajpendo.00059.2019. 31265325 10.1152/ajpendo.00059.2019

[CR52] Handschin C, Kobayashi YM, Chin S, Seale P, Campbell KP, Spiegelman BM. PGC-1α regulates the neuromuscular junction program and ameliorates Duchenne muscular dystrophy. Genes Dev. 2007;21:770–83. 10.1101/gad.1525107. 17403779 10.1101/gad.1525107 PMC1838529

[CR41] Hu CH, Yang CC, Tu SJ, Huang IJ, Ganbat D, Guo LY. Characteristics of the electrophysiological properties of neuromuscular motor units and its adaptive strategy response in lower extremity muscles for seniors with pre-sarcopenia: a preliminary study. Int J Environ Res Public Health. 2021;18:3063. 10.3390/ijerph18063063. 33809692 10.3390/ijerph18063063 PMC8002219

[CR26] Huang H, Zhao Y, Shang X, Ren H, Zhao Y, Liu X. CAIII expression in skeletal muscle is regulated by Ca2+-CaMKII-MEF2C signaling. Exp Cell Res. 2019;385:111672. 10.1016/j.yexcr.2019.111672. 31614133 10.1016/j.yexcr.2019.111672

[CR36] Jang YC, Van Remmen H. Age-associated alterations of the neuromuscular junction. Exp Gerontol. 2011;46:193–8. 10.1016/j.exger.2010.08.029. 20854887 10.1016/j.exger.2010.08.029 PMC3026920

[CR21] Joassard OR, Bélanger G, Karmouch J, Lunde JA, Shukla AH, Chopard A, et al. HuR mediates changes in the stability of AChR β-subunit mRNAs after skeletal muscle denervation. J Neurosci. 2015;35:10949–62. 10.1523/JNEUROSCI.1043-15.2015. 26245959 10.1523/JNEUROSCI.1043-15.2015 PMC6605275

[CR7] Larsson L, Degens H, Li M, Salviati L, Lee YI, Thompson W, et al. Sarcopenia: aging-related loss of muscle mass and function. Physiol Rev. 2019;99:427–511. 10.1152/physrev.00061.2017. 30427277 10.1152/physrev.00061.2017 PMC6442923

[CR2] Ling SM, Conwit RA, Ferrucci L, Metter EJ. Age-associated changes in motor unit physiology: observations from the Baltimore longitudinal study of aging. Arch Phys Med Rehabil. 2009;90:1237–40. 10.1016/j.apmr.2008.09.565. 19577038 10.1016/j.apmr.2008.09.565 PMC5496096

[CR9] Marzetti E, Calvani R, Lorenzi M, Marini F, D’Angelo E, Martone AM, et al. Serum levels of C-terminal agrin fragment (CAF) are associated with Sarcopenia in older hip fractured patients. Exp Gerontol. 2014;60:79–82. 10.1016/j.exger.2014.10.003. 25304331 10.1016/j.exger.2014.10.003

[CR10] Moreira-Pais A, Ferreira R, Oliveira PA, Duarte JA. A neuromuscular perspective of Sarcopenia pathogenesis: deciphering the signaling pathways involved. GeroScience. 2022;44:1199–213. 10.1007/s11357-021-00510-2. 34981273 10.1007/s11357-021-00510-2 PMC9213593

[CR29] Muñoz-Cánoves P, Scheele C, Pedersen BK, Serrano AL. Interleukin-6 myokine signaling in skeletal muscle: a double-edged sword? FEBS J. 2013;280:4131–48. 10.1111/febs.12338. 23663276 10.1111/febs.12338 PMC4163639

[CR39] Nishikawa H, Fukunishi S, Asai A, Yokohama K, Nishiguchi S, Higuchi K. Pathophysiology and mechanisms of primary Sarcopenia. Int J Mol Med. 2021;48:156. 10.3892/ijmm.2021.4989. 34184088 10.3892/ijmm.2021.4989

[CR38] Papke RL, Lindstrom JM. Nicotinic acetylcholine receptors: conventional and unconventional ligands and signaling. Neuropharmacology. 2020;168:108021. 10.1016/j.neuropharm.2020.108021. 32146229 10.1016/j.neuropharm.2020.108021 PMC7610230

[CR42] Pedersen BK, Fischer CPS. Beneficial health effects of exercise–the role of IL-6 as a myokine. Trends Pharmacol Sci. 2007;28:152–6. 10.1016/j.tips.2007.02.002. 17331593 10.1016/j.tips.2007.02.002

[CR48] Pelosi L, Berardinelli MG, Forcina L, Ascenzi F, Rizzuto E, Sandri M, et al. Sustained systemic levels of IL-6 impinge early muscle growth and induce muscle atrophy and wasting in adulthood. Cells. 2021;10:1816. 10.3390/cells10071816. 34359985 10.3390/cells10071816 PMC8306542

[CR50] Pototschnig I, Feiler U, Diwoky C, Vesely PW, Rauchenwald T, Paar M, et al. Interleukin-6 initiates muscle- and adipose tissue wasting in a novel C57BL/6 model of cancer-associated cachexia. J Cachexia Sarcopenia Muscle. 2023;14:93–107. 10.1002/jcsm.13109. 36351437 10.1002/jcsm.13109 PMC9891934

[CR57] Potthoff MJ, Arnold MA, McAnally J, Richardson JA, Bassel-Duby R, Olson EN. Regulation of skeletal muscle sarcomere integrity and postnatal muscle function by Mef2c. Mol Cell Biol. 2007;27:8143–51. 10.1128/MCB.01187-07. 17875930 10.1128/MCB.01187-07 PMC2169182

[CR20] Rudell JB, Ferns MJ. Regulation of muscle acetylcholine receptor turnover by β subunit tyrosine phosphorylation. Dev Neurobiol. 2013;73:399–410. 10.1002/dneu.22070. 23325468 10.1002/dneu.22070

[CR46] Schaap LA, Pluijm SM, Deeg DJ, Visser M. Inflammatory markers and loss of muscle mass (sarcopenia) and strength. Am J Med. 2006;119. 10.1016/j.amjmed.2005.10.049. 526.E9–526.E17. 10.1016/j.amjmed.2005.10.049 16750969

[CR35] Schaap LA, Koster A, Visser M. Adiposity, muscle mass, and muscle strength in relation to functional decline in older persons. Epidemiol Rev. 2013;35:51–65. 10.1093/epirev/mxs006. 23221972 10.1093/epirev/mxs006

[CR34] Schaap LA, van Schoor NM, Lips P, Visser M. Associations of sarcopenia definitions, and their components, with the incidence of recurrent falling and fractures: the Longitudinal Aging Study Amsterdam. J Gerontol Biol Sci Med Sci. 2018;73:1199–204. 10.1093/gerona/glx245. 10.1093/gerona/glx245 29300839

[CR17] Severinsen MCK, Pedersen BK. Muscle-organ crosstalk: the emerging roles of myokines. Endocr Rev. 2020;41:594–609. 10.1210/endrev/bnaa016. 32393961 10.1210/endrev/bnaa016 PMC7288608

[CR30] Silva KA, Dong J, Dong Y, Dong Y, Schor N, Tweardy DJ, et al. Inhibition of Stat3 activation suppresses caspase-3 and the ubiquitin-proteasome system, leading to preservation of muscle mass in cancer cachexia. J Biol Chem. 2015;290:11177–87. 10.1074/jbc.M115.641514. 25787076 10.1074/jbc.M115.641514 PMC4409274

[CR22] Soendenbroe C, Bechshøft CJL, Heisterberg MF, Jensen SM, Bomme E, Schjerling P, et al. Key components of human myofibre denervation and neuromuscular junction stability are modulated by age and exercise. Cells. 2020;9:893. 10.3390/cells9040893. 32268508 10.3390/cells9040893 PMC7226801

[CR27] Tanaka T, Narazaki M, Kishimoto T. Interleukin (IL-6) immunotherapy. Cold Spring Harb Perspect Biol. 2018;10:a028456. 10.1101/cshperspect.a028456. 28778870 10.1101/cshperspect.a028456 PMC6071487

[CR55] Taylor MV, Hughes SM. Mef2 and the skeletal muscle differentiation program. Semin Cell Dev Biol. 2017;72:33–44. 10.1016/j.semcdb.2017.11.020. 29154822 10.1016/j.semcdb.2017.11.020

[CR12] Tintignac LA, Brenner HR, Rüegg MA. Mechanisms regulating neuromuscular junction development and function and causes of muscle wasting. Physiol Rev. 2015;95:809–52. 10.1152/physrev.00033.2014. 26109340 10.1152/physrev.00033.2014

[CR47] Tsujinaka T, Fujita J, Ebisui C, Yano M, Kominami E, Suzuki K, et al. Interleukin 6 receptor antibody inhibits muscle atrophy and modulates proteolytic systems in interleukin 6 transgenic mice. J Clin Invest. 1996;97:244–9. 10.1172/JCI118398. 8550842 10.1172/JCI118398 PMC507086

[CR25] van Dijk M, Luiking Y, Dijk F, Jourdan M, Verlaan S, van Norren K. Loss of ex-vivo muscle function with preserved muscle mass in middle aged mice seems a sensitive indicator for the onset of Sarcopenia. Eur J Pharmacol. 2011;668:e19–20. 10.1016/j.ejphar.2011.09.238.

[CR24] van Dijk M, Nagel J, Dijk FJ, Salles J, Verlaan S, Walrand S, et al. Sarcopenia in older mice is characterized by a decreased anabolic response to a protein meal. Arch Gerontol Geriatr. 2017;69:134–43. 10.1016/j.archger.2016.11.014. 27918964 10.1016/j.archger.2016.11.014

[CR40] Verdijk LB, Snijders T, Beelen M, Savelberg HH, Meijer K, Kuipers H, et al. Characteristics of muscle fiber type are predictive of skeletal muscle mass and strength in elderly men. J Am Geriatr Soc. 2010;58:2069–75. 10.1111/j.1532-5415.2010.03150.x. 21054286 10.1111/j.1532-5415.2010.03150.x

[CR44] Visser M, Pahor M, Taaffe DR, Goodpaster BH, Simonsick EM, Newman AB, et al. Relationship of interleukin-6 and tumor necrosis factor-alpha with muscle mass and muscle strength in elderly men and women: the health ABC study. J Gerontol Biol Sci Med Sci. 2002;57:M326–32. 10.1093/gerona/57.5.m326. 10.1093/gerona/57.5.m326 11983728

[CR13] Wang X, McIntosh JM, Rich MM. Muscle nicotinic acetylcholine receptors may mediate trans-synaptic signaling at the mouse neuromuscular junction. J Neurosci. 2018;38:1725–36. 10.1523/JNEUROSCI.1789-17.2018. 29326174 10.1523/JNEUROSCI.1789-17.2018 PMC5815454

[CR23] Xie WQ, He M, Yu DJ, Wu YX, Wang XH, Lv S, et al. Mouse models of Sarcopenia: classification and evaluation. J Cachexia Sarcopenia Muscle. 2021;12:538–54. 10.1002/jcsm.12709. 33951340 10.1002/jcsm.12709 PMC8200444

[CR58] Yang B, Yang X, Sun X, Shi J, Shen Y, Chen R. IL-6 deficiency attenuates skeletal muscle atrophy by inhibiting mitochondrial ROS production through the upregulation of PGC-1α in septic mice. Oxid Med Cell Longev. 2022;2022:9148246. 10.1155/2022/9148246. 35528525 10.1155/2022/9148246 PMC9068301

[CR49] Yano T, Osanami A, Shimizu M, Katano S, Nagano N, Kouzu H, et al. Utility and safety of tocilizumab in Takayasu arteritis with severe heart failure and muscle wasting. ESC Heart Fail. 2019;6:894–7. 10.1002/ehf2.12487. 31297975 10.1002/ehf2.12487 PMC6676286

