# Supplementary material for: Acetylcholine receptor-β inhibition by interleukin-6 in skeletal muscles contributes to modulating neuromuscular junction during aging
Source: Mol Med. 2024 Oct 10;30:171. doi: 10.1186/s10020-024-00943-3 (PMC11468496; doi:10.1186/s10020-024-00943-3)

**Additional figures**

**Acetylcholine receptor-β inhibition by interleukin-6 in skeletal muscles contributes to modulating neuromuscular junction during aging**

Yanling Zhao^1†^, Han Yan^2†^, Ke Liu^2†^, Jiangping Ma^2^, Wenlan Sun^1^, Hejin Lai^3^, Hongli Li^1^, Jianbang Gu^4*^, He Huang^2,4*^

Affiliations

^1^ Department of Geriatrics, Shanghai General Hospital, Shanghai Jiao Tong University School of Medicine, Shanghai 200080, P.R. China

^2^ Department of Neurology, Tenth People's Hospital, Tongji University School of Medicine, Shanghai 200072, P.R. China

^3^ CAS Key Laboratory of Nutrition, Metabolism and Food Safety, Shanghai Institute of Nutrition and Health, University of Chinese Academy of Sciences, Chinese Academy of Sciences, Shanghai 200031, China.

^4^ Department of Neurology, Shanghai Tenth People's Hospital Chongming Branch, Shanghai 202150, P.R. China

^†^ These authors contributed equally.

***Corresponding authors:**

He Huang, Department of Neurology, Tenth People's Hospital, Tongji University School of Medicine, Shanghai 200072, P.R. China; Department of Neurology, Shanghai Tenth People's Hospital Chongming Branch, Shanghai 202150, P.R. China. E-mail: [morton162@hotmail.com](mailto:morton162@hotmail.com)

Jianbang Gu, Department of Neurology, Shanghai Tenth People's Hospital Chongming Branch, Shanghai 202150, P.R. China. E-mail: jiandor36@126.com

**Fig. S1** Interleukin **(**IL)-6 levels in the plasma increased during aging. **A**. Plasma IL-6 levels during the aging, as detected by enzyme-linked immunosorbent assay (ELISA). **B**. Serum IL-6 levels in early-aging mice after tocilizumab treatment. n=6. ***P* < 0.01. ****P*< 0.001.

**
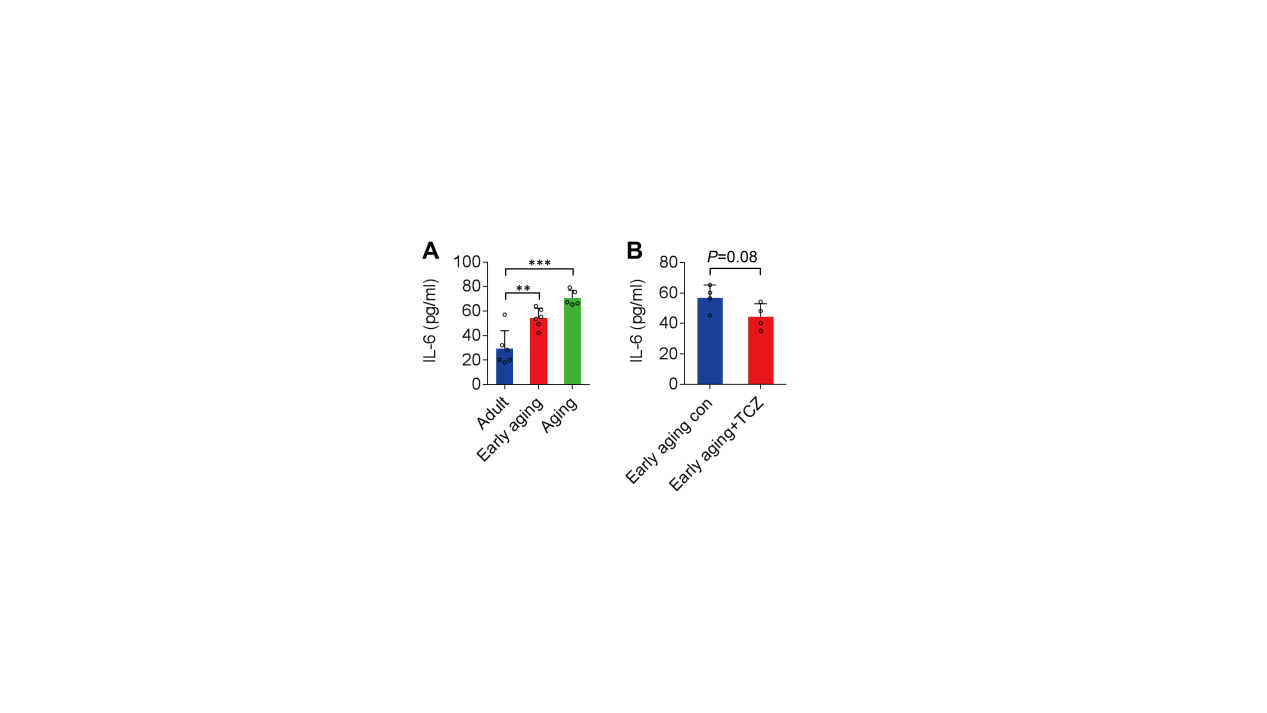
**

**Fig. S2** The myotubes obtained from C2C12 myoblast differentiation. **A.** Undifferentiated C2C12 myoblasts, **B.** differentiated C2C12 myotubes.

**
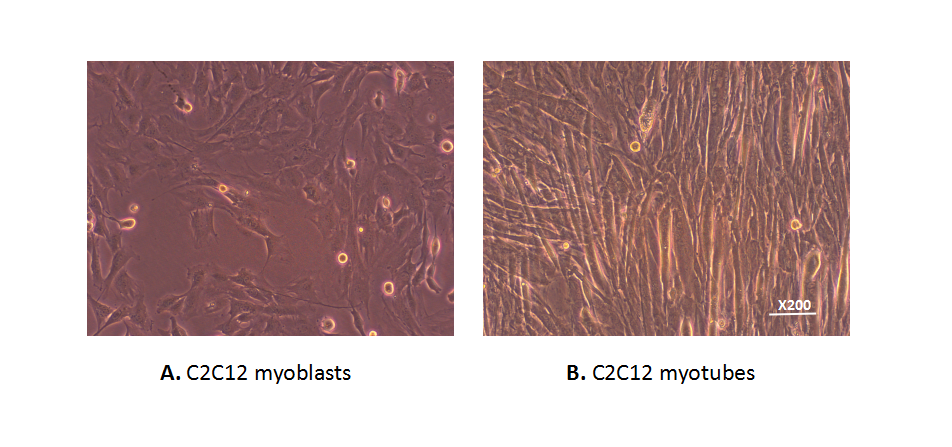
**

**Fig. S3** Peroxisome proliferator-activated receptor gamma coactivator 1-α **(**PGC1α) is involved in the regulation of acetylcholine receptor β-subunit (AChR-β) expression. AChR-β expression following administration of ZLN005 (10 μM, a PGC1α agonist) and SR18292 (10 μM, a PGC1α inhibitor). ***P* < 0.01. ****P*< 0.001.

**
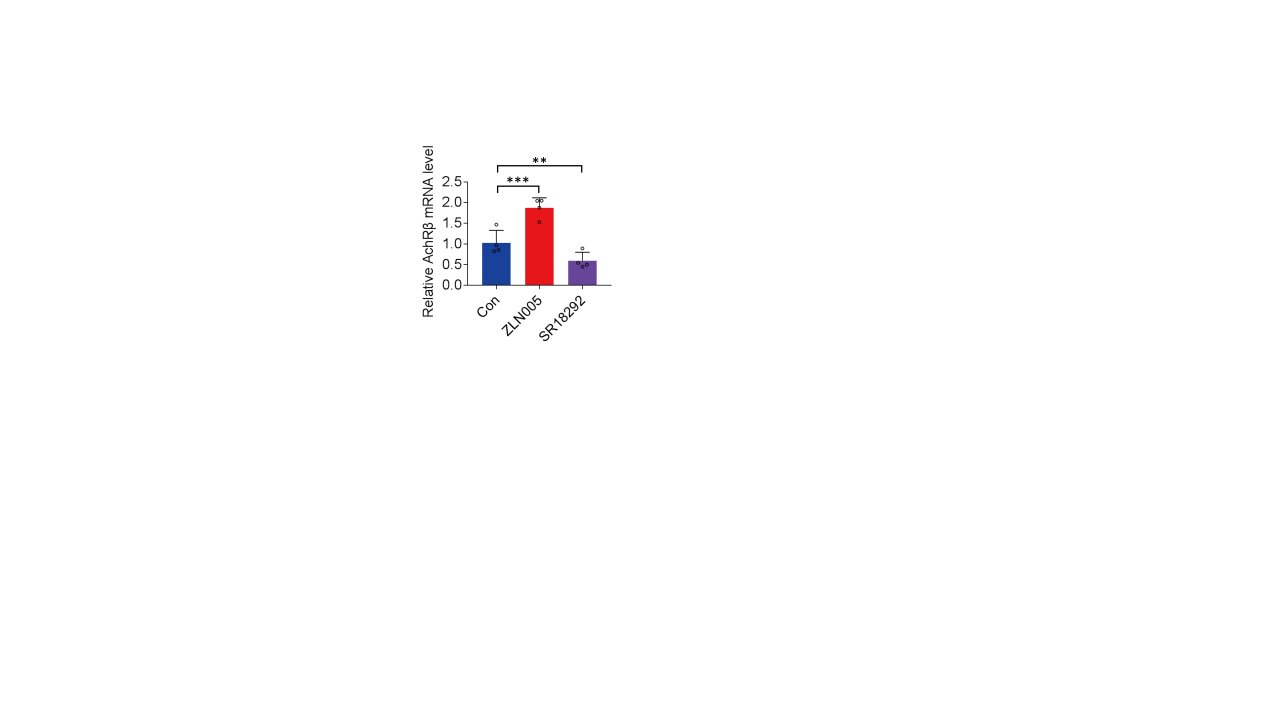
**

**Fig. S4** Plasmid construction. **A.** Three small interfering myocyte enhancer factor 2Cs **(**siMEF2Cs) were constructed and tested. **B.** The pcDNA3.1-MEF2C plasmid was constructed and tested. ***P* < 0.01. ****P*< 0.001.

**
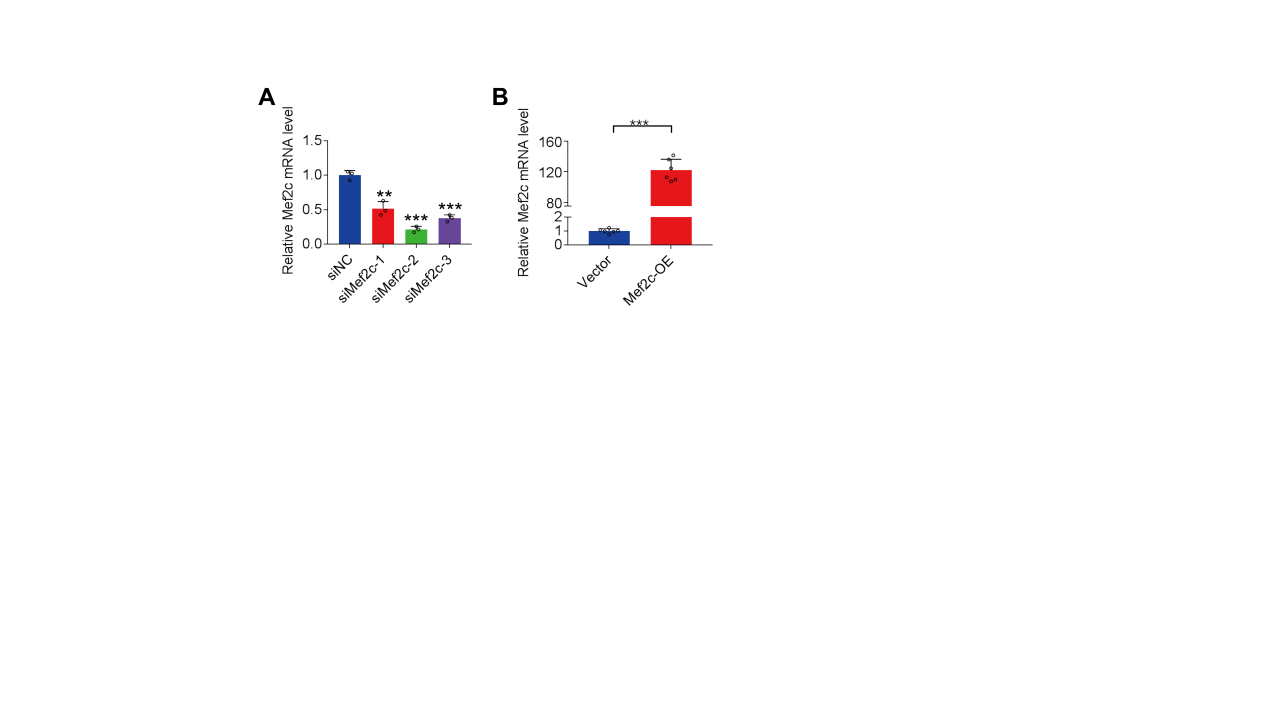
**

**Fig. S5** The acetylcholine receptor β-subunit (AChR-β) promoter. The predicted binding sites are marked in red.


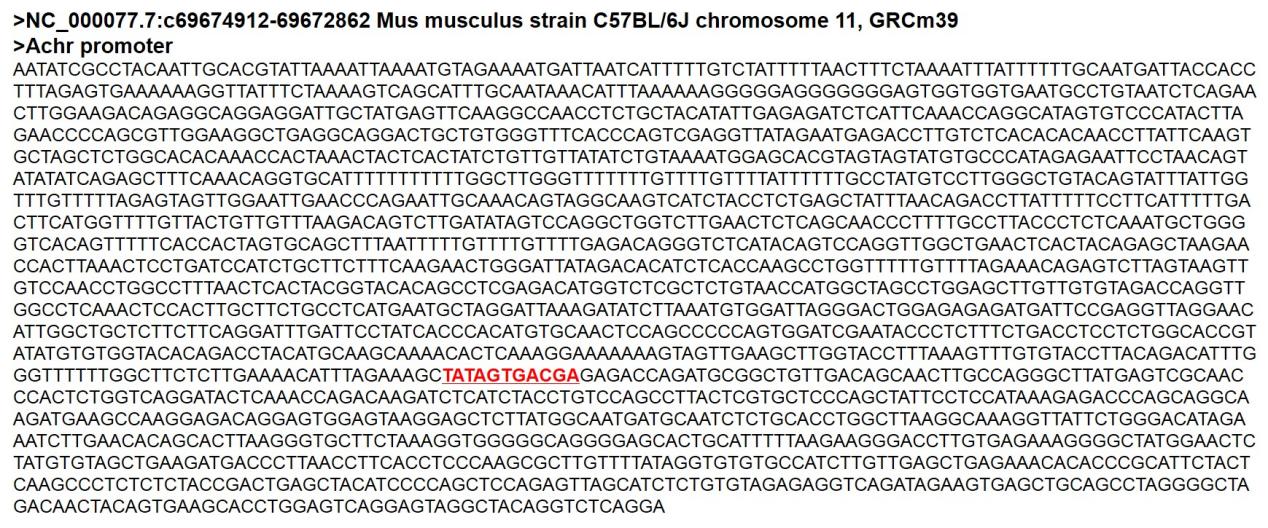


**Fig. S6** Interleukin (IL)-6, Peroxisome proliferator-activated receptor gamma coactivator 1-α **(**PGC1α), myocyte enhancer factor 2C (MEF2C), and acetylcholine receptor β-subunit (AChR-β) expression in aging C2C12 myoblasts. C2C12 cells were incubated with 50 mM C2 ceramide (HY-101180; MedChemExpress) for 8 h to induce senescence [Mech. Ageing Dev. 2013, 134, 548–559]. **A.** C2C12 myotubes were analyzed using β-galactosidase staining. Senescence-accelerated myotubes were stained with β-galactosidase staining kit (C0602; Beyotime) according to the manufacturer’s instructions. **B** **and** **C**. The gene and protein expression of IL-6 increased, whereas that of PGC1α, MEF2C, and AChR-β decreased in senescence-accelerated myotubes. n=3. **P* < 0.05. ***P* < 0.01. ****P*< 0.001.


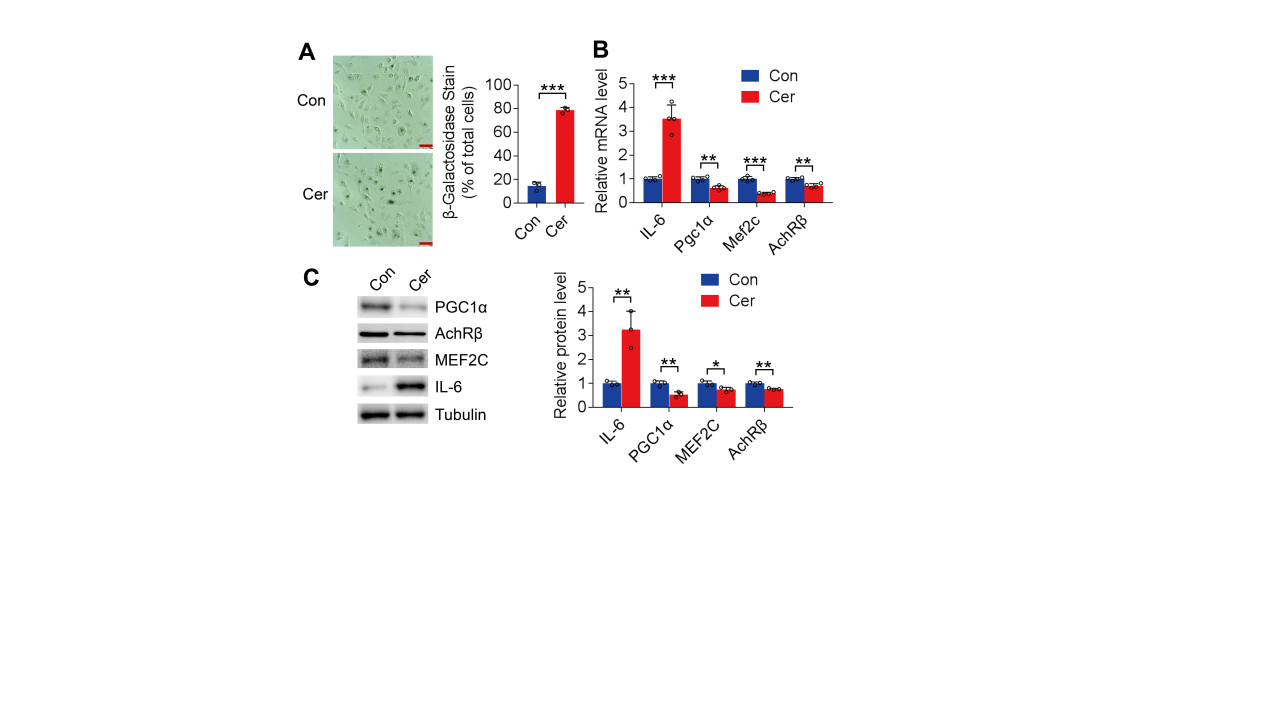

Supplement: Supplementary file 1 — Supplementary Material 1 [file 10020_2024_943_MOESM1_ESM.docx]
